# Modeling and MEG evidence of early consonance processing in auditory cortex

**DOI:** 10.1371/journal.pcbi.1006820

**Published:** 2019-02-28

**Authors:** Alejandro Tabas, Martin Andermann, Valeria Schuberth, Helmut Riedel, Emili Balaguer-Ballester, André Rupp

**Affiliations:** 1 Max Planck Institute for Human Cognitive and Brain Sciences, Leipzig, Germany; 2 Faculty of Science and Technology, Bournemouth University, Poole, United Kingdom; 3 Section of Biomagnetism, Department of Neurology, Heidelberg University Hospital, Heidelberg, Germany; 4 Bernstein Center for Computational Neuroscience, Heidelberg/Mannheim, Mannheim, Germany; Istituto Superiore Di Sanita, ITALY

## Abstract

Pitch is a fundamental attribute of auditory perception. The interaction of concurrent pitches gives rise to a sensation that can be characterized by its degree of consonance or dissonance. In this work, we propose that human auditory cortex (AC) processes pitch and consonance through a common neural network mechanism operating at early cortical levels. First, we developed a new model of neural ensembles incorporating realistic neuronal and synaptic parameters to assess pitch processing mechanisms at early stages of AC. Next, we designed a magnetoencephalography (MEG) experiment to measure the neuromagnetic activity evoked by dyads with varying degrees of consonance or dissonance. MEG results show that dissonant dyads evoke a pitch onset response (POR) with a latency up to 36 ms longer than consonant dyads. Additionally, we used the model to predict the processing time of concurrent pitches; here, consonant pitch combinations were decoded faster than dissonant combinations, in line with the experimental observations. Specifically, we found a striking match between the predicted and the observed latency of the POR as elicited by the dyads. These novel results suggest that consonance processing starts early in human auditory cortex and may share the network mechanisms that are responsible for (single) pitch processing.

## Author summary

In this work, we argue that human auditory cortex processes pitch and consonance by means of a common neural network mechanism operating at early cortical stages. We introduce a neural population model of cortical pitch processing that contains biophysically realistic synaptic and neural parameters. The model quantitatively explains, for the first time, the neuromagnetic responses observed in human auditory cortex during pitch perception. The model is subsequently used to elucidate the cortical processing of musical dyads, in which concurrent pitches lead to the perception of consonance or dissonance. Interestingly, the model predicts that sounds perceived as dissonant need more time for cortical processing than consonant sounds. This prediction is experimentally validated by recording cortical neuromagnetic fields in response to consonant and dissonant dyads. Taken together, our results suggest a novel mechanistic explanation for early cortical processing of musical harmony, in the sense that the differential response to consonance and dissonance starts early, simultaneously to (single) pitch processing, in auditory cortex.

## Introduction

Pitch is the perceptual correlate of the periodicity in a sound’s waveform, and thus a fundamental attribute of auditory sensation. It forms the basis of both music and speech perception. However, understanding pitch processing as elicited by concurrent sounds in human auditory cortex is still a major challenge in auditory neuroscience [1–5].

A combination of two sounds that simultaneously elicits two different pitches is called a dyad, and the pitch interactions within the dyad give rise to a sensation that can be characterized by its *consonance* or *dissonance*. Loudness, timbre, and the fundamental periodicities of the two sounds can have subtle effects on whether a dyad is perceived as consonant or dissonant. However, the dominant factor in determining the degree of a dyad’s consonance is the relationship between the fundamental periods of the sounds that make up the dyad: simple periodicity ratios result in more consonant sensations. In contrast, the sensation becomes more and more dissonant as the complexity of the periodicity ratio increases [6, 7]. It has been previously proposed that dissonance correlates with the beating or *roughness* sensation that is elicited by the interfering regularities of the dyad components [6, 7]. However, listeners who showed impaired pitch perception but were sensitive to beating and roughness were unable to differentiate between consonant and dissonant dyads [1, 8]. This suggests that pitch- rather than roughness-related auditory processing is responsible for consonance perception.

Neurophysiological evidence for a close link between consonance and pitch has recently been provided by Bidelman and colleagues [2]. Their study showed, using electroencephalography (EEG), that the amplitude of the cortical pitch onset response (POR) is strongly modulated by a dyad’s perceived consonance. The POR is a pitch-selective component of the transient auditory evoked potential/field (AEP/AEF) that occurs within the time range of the well-known N100 deflection, around 100 ms after pitch onset [9]. The morphology of the POR is strongly correlated with the perceived pitch in single tones: its latency scales linearly with the period of the sound and its amplitude increases with the strength of the pitch percept [9, 10]. The neural sources of the POR are located in the anterolateral section of Heschl’s gyrus (alHG) in auditory cortex [2, 9], in agreement with the anatomical location of pitch-selective neurons in non-human primates (e.g., [11–13]), and with pitch-selective regions that were reported for human listeners [14–18].

Further experiments in human subjects demonstrated that the dyad-evoked frequency-following response in the brainstem is predictive for the perceived consonance of a dyad (for a review, see [19]). However, functional magnetic resonance imaging (fMRI) studies showing selective activation to consonance/dissonance contrasts in the superior temporal gyrus [20] and in frontal cortex [21] led the auditory community to link neural representations of consonance and dissonance with higher cognitive processes [22].

In this study, we used a combined experimental and theoretical approach to assess whether consonance and pitch share similar processing mechanisms in human auditory cortex. Towards this goal, we first developed an ensemble model of cortical pitch responses, specifically designed to understand the mesoscopic representation of pitch in alHG. The model can account, mechanistically, for the POR latency effects that have been reliably reported in numerous experimental settings [9] but remained poorly understood. Second, we recorded the AEF elicited by consonant and dissonant dyads using magnetoencephalography (MEG). Our experimental results revealed a strong correlation between the POR latency and the degree of consonance, extending previous EEG findings [2]. Finally, we aimed to replicate the results from the MEG experiment using our model. If the hypothesis that consonance and pitch are processed by similar mechanisms in cortex is correct, we would expect the model to explain the dependence of POR latency on the degree of consonance *without* the inclusion of higher processing stages within the auditory hierarchy [20, 21]. In line with this hypothesis, the model provides a quantitative explanation for the relationship between the POR dynamics and consonance, suggesting that consonance and dissonance perception might be linked to pitch processing regions in auditory cortex, prior to higher-order processing.

## Results

### Neural mechanisms underlying pitch processing in auditory cortex

#### Model overview

We introduced a model of cortical pitch processing designed to understand the morphology of the cortical response to pitch onset (see full description in Methods). The model consists of three processing stages located at different levels of the auditory hierarchy. In the first stage, an array of idealized coincidence detector units extracts periodicities from the auditory nerve activity in response to the target stimulus [23, 24]. The second and third stages, putatively located at adjacent locations of alHG, transform the output of the periodicity detectors into a stable representation of pitch.

**Fig 1:**
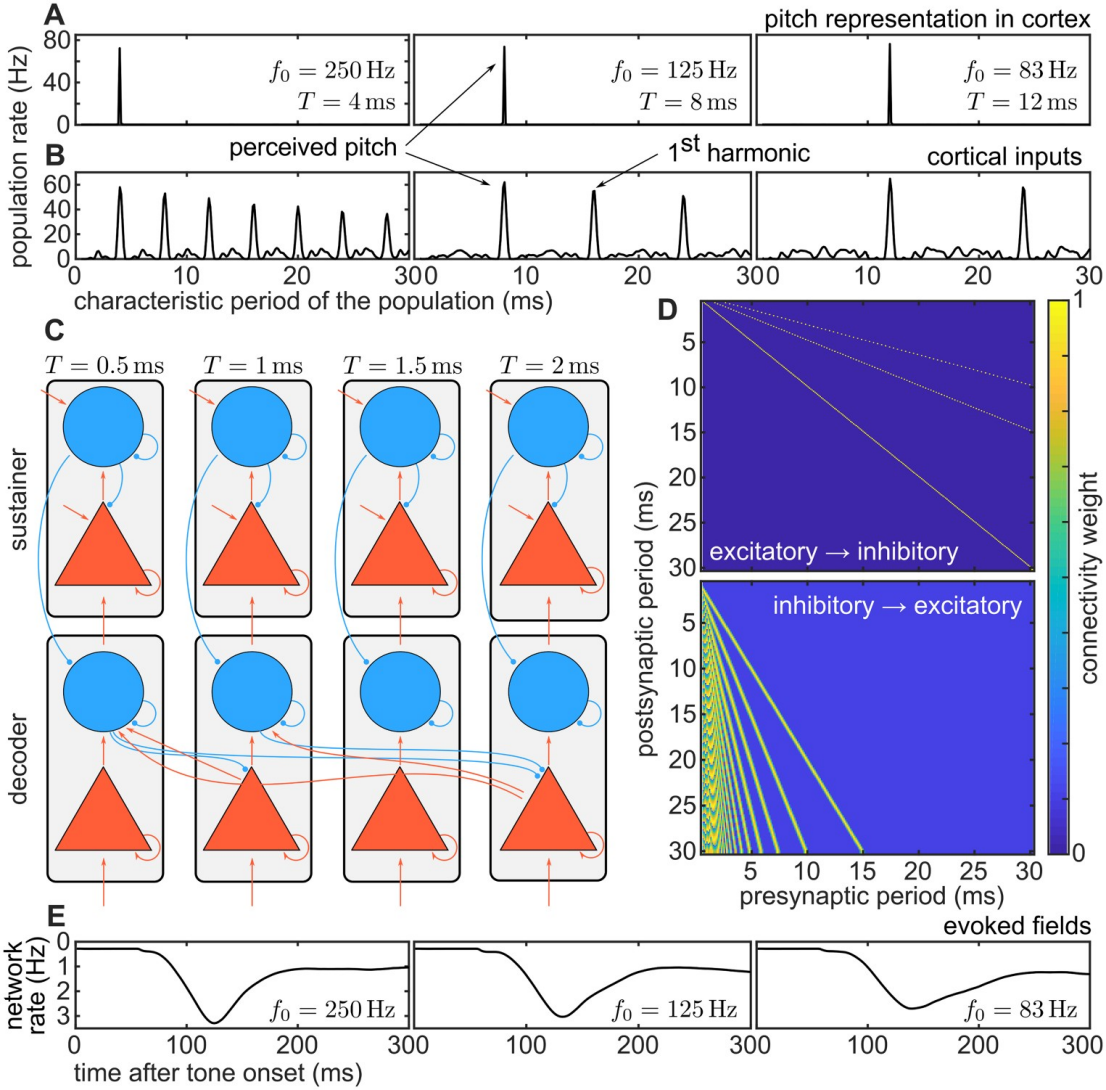
Basic schematics of the model. Architecture (C, D) and responses (A, B, E) of the model to three stimuli with different pitches. Stimulus used to produce the examples were iterated rippled noises with 16 iterations, bandpass filtered between 0.8 and 3.2 kHz, with three fundamental periods *T* = 4, 8, and 12 ms, corresponding to the three columns of the figure. (A) Excitatory *population rate* in the decoder (i.e., the time-average response for each of the excitatory ensembles in the decoder). The rate was averaged between 250 and 300 ms after the sound onset. The main peak of the population rate at the decoder represents stimulus pitch. (B) Excitatory *population rate* of the cortical input (i.e., the time-average response for each periodicity detector). As in panel A, the rate was averaged between 250 and 300 ms after sound onset. The first peak in this representation corresponds to the fundamental period of the stimulus; subsequent peaks correspond to its lower harmonics. (C) Model architecture. The model consists of two networks, each with 250 columns (grey rectangles). Each column comprises an excitatory (triangle) and an inhibitory (circle) ensemble, and represents a specific pitch value ranging from 1/(0.5 ms) = 2 kHz to 1/(30 ms) = 33.3 Hz. The bottom network is termed the *decoder*, and the top network is called the *sustainer* (see text). Red arrows between ensembles represent excitatory connections; blue lines ended in a circle denote inhibitory connections. (D) Connectivity weights between excitatory and inhibitory ensembles in the decoder network. (E) Decoder’s *network rate* (i.e., the average response across all the excitatory ensembles of the decoder network at each instant *t*), monotonically related to the auditory evoked fields. The *y*-axis was inverted for consistency with the standard representation of the evoked fields. The network rate peak latency correlates with the latency of the pitch onset response.

Auditory nerve responses were generated by a recent biophysical model of the auditory periphery [25, 26], followed by a standard periodicity detection process [23, 24, 27, 28]. At this stage, the stimulus representation typically shows a well-known harmonic structure along the periodicity axis [28], with prominent peaks of activation at the neurons which encode the pitch of the stimulus and its lower harmonics (see Fig 1E).

The array of periodicity detectors provides excitatory input to a first cortical processing stage, termed the *decoder* network in this study. The *decoder* network is putatively located in alHG and effectively decodes the pitch value(s) from the subcortical input. The decoder network connects to a second cortical ensemble network, termed *sustainer* network; this stage integrates the output of the decoder network and top-down modulates it through cortico-cortical efferents, in a mechanism that is reminiscent to recent models of perceptual decision making [29].

Both decoder and sustainer comprise a network of cortical microcolumns, each of which is tuned to a specific pitch along the human perceptual range (see Methods for details). In the model, pitch is coded in the active pitch-selective populations of the processing network (Fig 1B), in agreement with various neuroimaging experiments that indentified alHG as a candidate region for the processing of pitch information in auditory cortex [9, 12, 18, 30–35]).

**Fig 2:**
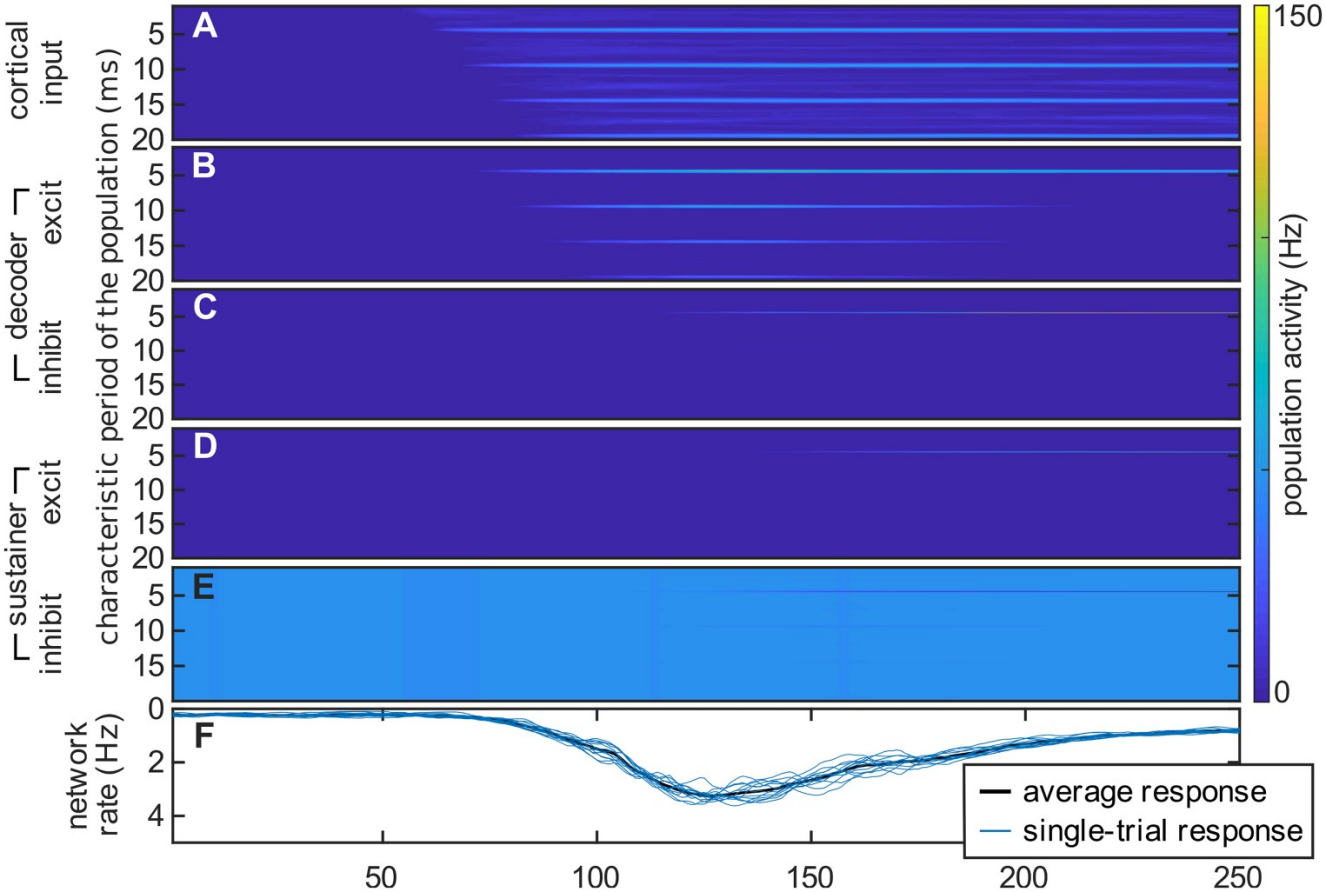
Illustration of the decoding process. The plots show the evolution of rate variables of the model during the processing of an iterated rippled noise with a fundamental period of *T* = 5 ms (parameters were as in Fig 1). (A–E) Evolution of the neural ensembles encoding characteristic periods between 0.5 ms and 20 ms. (A) Activity of periodicity detectors within the first stage of the model. (B, C) Activity of excitatory and inhibitory ensembles in the *decoder* network. (D, E) Activity of excitatory and inhibitory ensembles in the *sustainer* network. (F) Aggregated excitatory activity in the decoder (*y*-axis was inverted like in Fig 1A). Detailed dynamics of the process are illustrated in S1 Video.

Microcolumns in the cortical networks are modeled as blocks comprising an excitatory and an inhibitory neural ensemble (Fig 1C), which communicate with each other through realistic synapses. Connectivity weights between populations in the decoder network (Fig 1D) are specifically tuned to facilitate the inhibition of the periodicity detectors representing lower harmonics (Fig 2, see also S1 Fig). Similar connectivity patterns have been reported in the mammalian auditory cortex [36, 37]; moreover, neurons mapping harmonic templates to a pitch-selective representation like those introduced in this model have been recently reported in the primate auditory cortex [13].

The model enables us to perform quantitative predictions of the cortical response elicited by the pitch of a stimulus. Specifically, the equivalent dipole moment should be monotonically related to the global excitatory response of the decoder network (see Methods for details), while the characteristic period of the excitatory population with the largest activity decodes the perceived pitch (see for instance Fig 1B). We hypothesize that the decoding mechanism is responsible for the dynamics of the POR in human auditory cortex.

#### Dynamics of the decoder network

Fig 2 illustrates an example of the model dynamics in response to a stimulus with a pitch corresponding to *T* = 5 ms (i.e., *f* = 200Hz, more details are shown in S1 Video). In a first step, periodicity detectors, tuned to *T* ≃ 5 ms, become active after *t*_1_ ∼ 1.25 *T* [38] (see the top prominent horizontal line at *T* = 5 ms in Fig 2A); these populations provide bottom-up excitatory input to the excitatory ensemble in the corresponding decoder network column (see Fig 2B). Likewise, the harmonics of the stimulus pitch period (i.e., 2 *T*, 3 *T*, etc) are subsequently represented in the periodicity detectors after *t*_2_ = 2*t*_1_, *t*_3_ = 3*t*_1_ etc., and provide the input to the corresponding excitatory populations in the decoder network (see Fig 2B).

Excitatory ensembles characterized by the periods {*T*, 2 *T*, 3 *T*…} are connected to the inhibitory population characterized by the fundamental period of such series, *T* (see Fig 1). Synaptic efficacy is tuned such that the inhibitory drive is strong enough only when a sufficient number of excitatory inputs are simultaneously active. The conductivity between excitatory and inhibitory ensembles in the decoder is tuned within a realistic range such that three harmonic inputs are necessary to activate each inhibitory ensemble change:l145a. For instance, the inhibitory population characterized by *T* ≃ 5 ms in Fig 2C becomes active only when it receives simultaneous synaptic input from excitatory ensembles characterized by the periods *T* = 5 ms, 2*T* = 10 ms, and 3*T* = 15 ms.

Correspondingly, the inhibitory ensemble associated with the period *T* is connected to excitatory populations encoding the lower harmonics {2 *T*, 3 *T*, 4 *T*, …} (see Fig 1). When sufficiently active, it progressively silences excitatory populations that do not correspond to the fundamental period of the stimulus (see in Fig 2B an example of this shunting process in the decoder excitatory network between *t* = 120 ms and *t* = 200 ms).

This process illustrates a possible mechanism underlying the dynamics of the cortical pitch onset response: first, the accumulation of excitatory activity in the decoder results in the progressive increase of the simulated field magnitude observed between *t* = 75 ms and *t* = 130 ms in Fig 2F. Second, the subsequent decay of the collective excitatory response between *t* = 120 ms and *t* = 200 ms in the figure is caused by the action of the most activated inhibitory ensemble on all excitatory populations encoding the lower harmonics of the stimulus’ fundamental period, *T*. We identify the maximum in the aggregated excitatory activity, corresponding to the time point in which the model performs a decision about the pitch of the stimulus, with the POR latency (further details regarding these dynamics are shown in S1 Video and in S1 Fig).

Thus, in this model, the linear dependence of the POR latency with single-pitch IRN stimuli is implemented by the decoding process. Since a periodicity detector takes around 1.25*T* to detect a pitch of periodicity *T*, this same mechanism could be responsible for the minimum stimulus duration required for robust pitch discrimination, which is around four times the period of the stimulus [9].

#### Dynamics of the sustainer network

**Fig 3:**
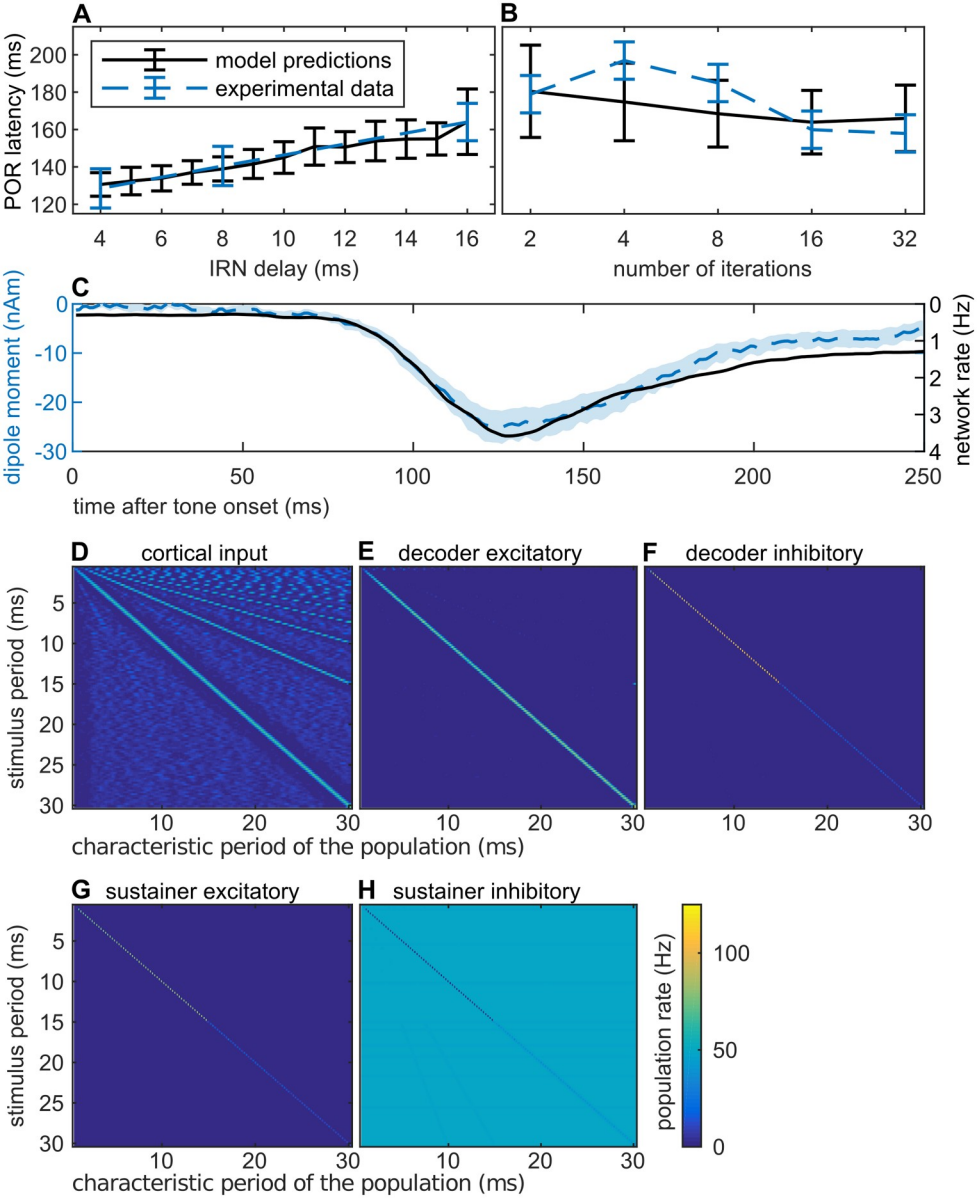
Model responses to single IRNs. (A, B) Latency predictions for iterated rippled noise compared with experimental data reported by a previous study [9]. Simulations were performed using the same stimuli parameters as in the original experiment (i.e., (A) 16 iterations, (B) 16 ms delay; both bandpass filtered between 0.8 kHz and 3.2 kHz). Latency predictions were averaged across *N* = 60 runs of the model, error bars are standard errors of the mean. (C) Comparison of the collective response of the excitatory ensembles in the decoder (computed as an average across populations) with the equivalent dipole moment elicited at the POR generator. The stimulus was an iterated rippled noise with 16 iterations and a delay of 8 ms, bandpass filtered between 0.8 kHz and 3.2 kHz. Shaded contours are standard errors. (D–H) Averaged responses at different stages of the model: (D) periodicity detectors, (E/F) excitatory/inhibitory ensembles in the decoder, (G/H) excitatory/inhibitory ensembles in the sustainer.

The dynamics of the decoder network suffice to explain how pre-cortical representations (Fig 1E) are transformed into the cortical pitch response (Fig 1A and 1B). However, after the transformation has taken place, the excitatory ensembles corresponding to the lower harmonics of the stimulus pitch are no longer active, and hence the inhibitory population silencing them loses its drive. Therefore, without top-down control, the decoder network would rapidly reset and repeatedly attempt to decode the pitch, eliciting a series of PORs. This, however, does not reflect the experimental observations (Fig 3C). The role of the sustainer network is thus to regulate the dynamics of the decoder network to effectively *sustain* the previously decoded value, until a significant change is produced in the cortical input.

In the absence of external input, the sustainer network rests at equilibrium, with a steady activation in the inhibitory populations and complete deactivation of the excitatory populations (Fig 2D–2E). Excitatory/inhibitory ensembles in the sustainer receive direct bottom-up input from their respective excitatory/inhibitory counterparts in the decoder (Fig 1D). Thus, a significantly active inhibitory population in the decoder effectively silences the analogous inhibitory population in the sustainer. If this afferent drive coincides with a strong activation of one of the excitatory populations in the decoder (for instance, the one characterized by a period *T* = 5 ms in Fig 2B), the combined bottom-up input results in a strong activation of the equivalent excitatory population in the sustainer (see Fig 2D).

Simultaneously, top-down efferents connect each excitatory population in the sustainer with its inhibitory counterpart in the decoder network (Fig 1C), compensating for the loss of excitatory drive in the silenced populations for as long as the subcortical input remains unchanged. The behavior of the network during pitch changes is described in detail in S2 Fig.

#### Model responses to single IRNs

The POR is defined as the subcomponent of the N100 transient that responds selectively to pitch onset and pitch changes [9]. In order to isolate the POR from other subcomponents of the N100 like the energy onset response, previous MEG studies used iterated rippled noise (IRN) preceded by a noise burst of the same energy and bandwidth [2, 9]. The POR is then measured as the transient elicited at the transition between noise and IRN; i.e., at the onset of pitch. Thus, we used IRN stimuli with different pitch values to tune and test the behaviour of our model (an isolated POR can only be elicited using energy-balanced stimuli such as noise-matched IRN; but see also S4 Fig for subsequent predictions drawn for pure tones [39]).

Model latencies for the POR elicited by IRN stimuli are compared with experimental data in Fig 3A and 3B. Results show that the model reproduces the relation between the POR latency and the period of the stimuli as typically reported in the MEG literature [9]. Results for other IRN stimuli using different parametrizations are shown in S3 Fig. This faithful representation of the pitch/latency relationship is a direct consequence of the model parameters tuning, that makes the model to require three input harmonics to perform a perceptual decision on the IRN pitch (see previous section and Methods). However, the co-dependency of the collective decoder response and the AEF is not an obvious result of model tuning.

We observed a systematic discrepancy in the latest section of the predicted and observed AEF magnitude for some of the tested stimuli (see an example in Fig 3C). Two factors could explain these difference: first, we assumed that the model collective activation and its derived equivalent dipole are linearly related [40], but the actual dependence between neural activity and the evoked fields depends on the relative orientation of the cortical columns, which is unknown. Thus, we cannot draw exact predictions on the absolute magnitude of the AEFs evoked by the model ensembles. Second, although the AEF depicted in Fig 3C corresponds to a fit of the POR response, dipole fittings were performed within a 30 ms window centered at the POR peak. Thus, it is possible that the final portion of the AEF time series might be contaminated by later components such as the P200. Similar disagreements between the AEFs and the collective activity of the decoder will be observed in the model predictions for the dyads.

To test whether the pitch of the IRN stimuli is correctly encoded in the model, we plotted the average activation in the different ensembles (Fig 3D–3H). Neither periodicity detectors nor excitatory ensembles of the decoder network show salient pitch selectivity; however, the decoded pitch is observed clearly both in the inhibitory populations of the decoder network and in the sustainer network. Fig 3, S3 and S5–S7 Figs indicate that the model can eventually decode the pitch of the stimulus when at least two harmonics are present at the cortical input (since we only consider periodicity detectors tuned to periods below *T*_max_ = 30 ms, the highest period robustly extracted by the model is *T*_max_/2 = 15 ms; a larger pitch range could be easily achieved by increasing *T*_max_). Robust decoding for IRN stimuli with different parametrizations, pure tones, harmonic complex tones (including virtual pitch [41]), and click trains [42] is shown in S3 and S5–S7 Figs.

### Neuromagnetic correlates of consonance and dissonance in auditory cortex

**Fig 4:**
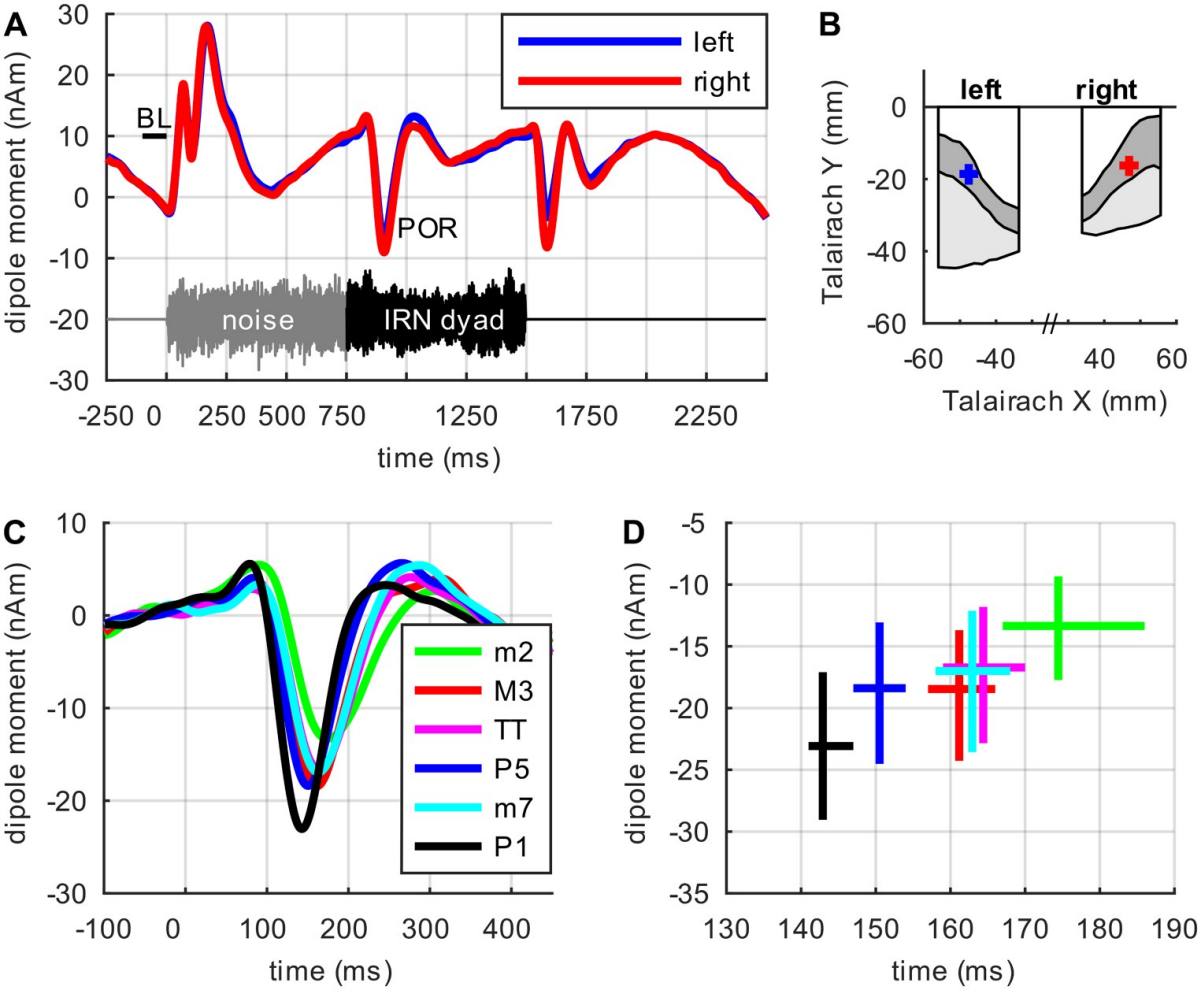
Auditory fields evoked at dyad onset. (A) MEG grand-mean source waveforms in response to the pooled stimulus conditions. The course of the stimuli is shown in grey (noise) and black (IRN) below the source waveforms; note the prominent negative POR deflection (N1m) at the transition from the first to the second stimulus segment. BL = baseline. (B) Projection of the dipole locations (means and 99% bootstrap confidence intervals) onto the axial view of auditory cortex as suggested by Leonard et al. [43]. (C) Morphology of the POR in response to the dyad onset in the single experimental conditions (second stimulus segment), pooled over hemispheres. (D) 99% Bootstrap confidence intervals for the POR amplitudes and latencies in the single experimental conditions. In subplots (B, D) confidence intervals are bias-corrected and accelerated to compensate for bias and skewness in the distribution of the bootstrap estimates, as recommended by Efron and Tibshirani [44].

Next, we recorded neuromagnetic fields evoked by six different dyads from 37 normal hearing subjects. Data were preprocessed using standard MEG procedures and equivalent current dipoles were fitted for the POR, independently in each subject and hemisphere, and pooled over conditions (see Methods). Dipole locations in Talairach space are plotted in Fig 4B.

Dyads consisted of two IRN sounds. The lower note pitch was 160 Hz; the pitch of the upper note was adjusted accordingly to form either a consonant dyad (unison, P1; perfect fifth, P5; major third, M3) or a dissonant dyad (tritone, TT; minor seventh, m7; minor second, m2). To dissociate the energy onset response in planum temporale from the POR in alHG, the dyads were preceded by an energy-balanced noise segment, cross-faded with the dyad to avoid discontinuous waveforms (like for the single IRN sounds analyzed in the previous section; see Methods).

Fig 4A presents the MEG grand-mean source waveforms, for both hemispheres, in response to the six stimulus conditions. The noise onset from silence (depicted in grey below the source waveforms) was followed by a transient P1m-N1m-P2m AEF complex. Since the first stimulus segment did not vary between conditions, we did not expect to find any significant differences in the corresponding neuromagnetic activity at this point.

In contrast, the transition to the second stimulus segment (IRN dyads, black signal below the source waveforms) elicited prominent POR waves and the morphology of the POR varied considerably between conditions. Fig 4C shows close-up views of the POR. Consonant dyads (pooled conditions [P1+P5+M3]) elicited a much earlier (*p* <.0001) and larger (*p* <.0001) POR than dissonant dyads (pooled conditions [m7+TT+m2]). Fig 4D depicts 99% bootstrap confidence intervals for the POR amplitudes and latencies pooled over hemispheres in response to the experimental conditions; the activity pattern observed here also points to a close relationship between the degree of a dyad’s consonance and the morphology of the respective POR.

When pooling across conditions, we found a difference between the left and the right hemisphere in the POR amplitude (*p* = .01), but not in the POR latency (*p* = .36); also, the difference between the neuromagnetic responses to consonant or dissonant dyads did not significantly vary between hemispheres (latency: *p* = .58, amplitude: *p* = .48).

### Neural mechanisms underlying the responses of auditory cortex to consonance and dissonance

The POR latency difference in response to consonant and dissonant dyads in alHG suggests that consonance and dissonance are computed at relatively early stages of the cortical auditory hierarchy. We used our model of cortical pitch processing, designed to reproduce the neuromagnetic responses elicited by iterated rippled noises, to test this interpretation. If the differential responses to consonance and dissonance in alHG were intrinsic to pitch processing, we would expect our model to be able to reproduce this behavior.

**Fig 5:**
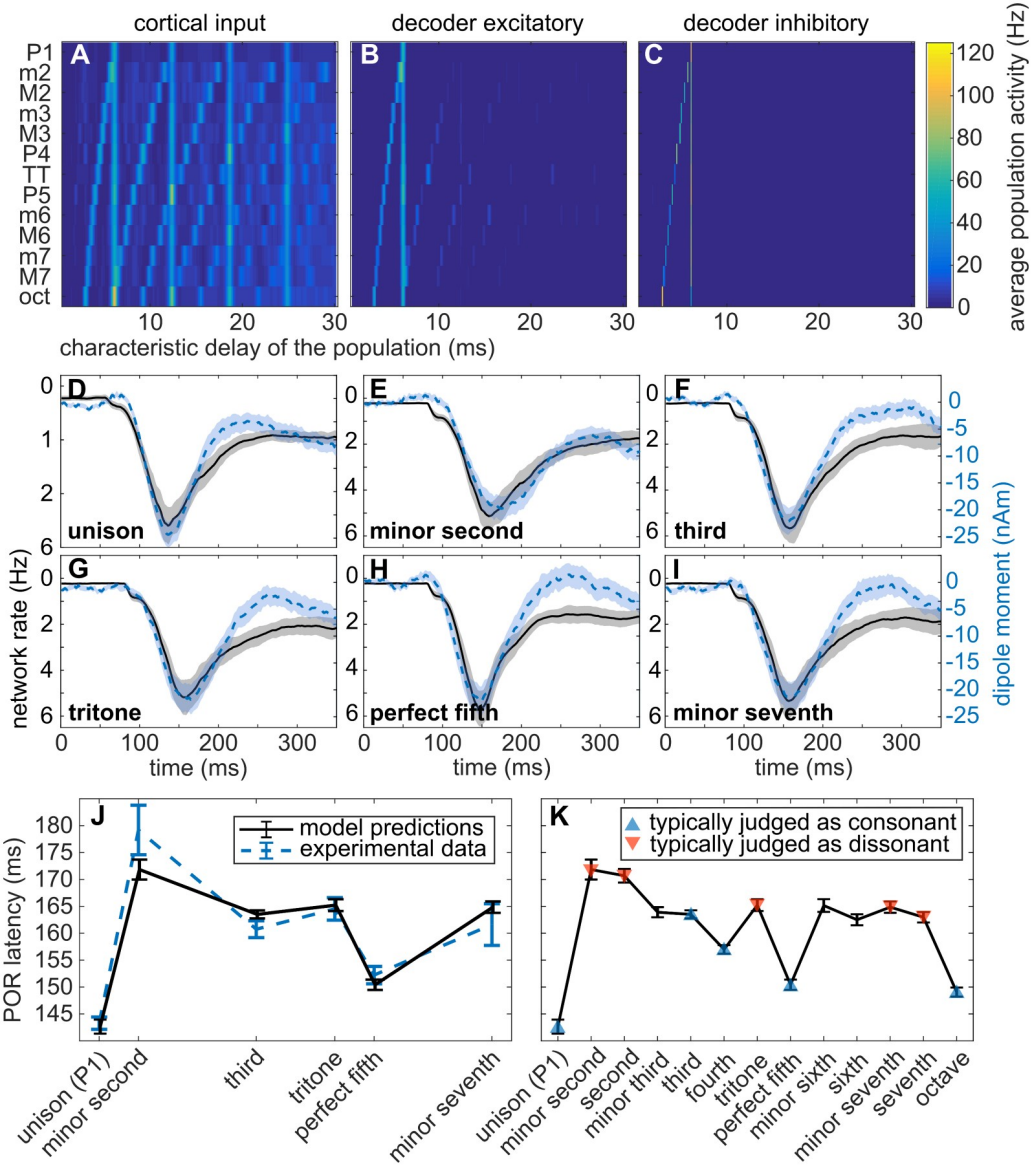
Model responses to the IRN dyads. (A–C) Neural representation of the dyads at different stages of the model: (A) periodicity detectors, (B/C) excitatory/inhibitory ensembles in the decoder network; each row shows the activity elicited by each dyad. Excitatory and inhibitory ensembles in the sustainer are precisely correlated with the decoder-inhibitory heatmap. (D–I) Examples of the collective excitatory activity in the decoder network (monotonically related to the equivalent dipole moment elicited by the network) in comparison with the elicited dipole moment measured during the experimentation in the neural generator of the POR. The scale of the field derived for the unison dyad was adjusted to account for the comparatively smaller effect on the network of the unison input, which effectively activates half of the populations than the other dyads. (J) Latency predictions for IRN dyads compared with the experimental results reported in the previous section. (K) Latency predictions for all dyads in the chromatic scale. Consonant dyads are represented with a green triangle, whilst strongly dissonant dyads are represented with a red triangle; dissonance was assessed according to Helmholtz [6] (see table in Fig 61 of the original text). Model predictions were averaged across *N* = 60 runs, error bars and shaded contours are standard errors. Blue shaded contours correspond to the experimental observations; grey shaded contours correspond to the model simulations.

First, we verified that the model was able to provide a joint representation of the two pitches comprised in the dyads; the results are shown in Fig 5A–5C. As in Fig 3, the plots show the average activation of the different ensembles (*x*-axis) for the 13 dyads in the chromatic scale (*y*-axis). The vertical line in Fig 5C indicates the first note common to all dyads; the diagonal the neural representations of the second notes. It should be emphasized that even phenomenological (biologically inspired but with low realism) models of pitch perception are generally unable to decode sounds with concurrent pitches (e.g., [45, 46]; see [47] for a review).

Fig 5J shows the latency predictions of the model and the experimental data for the respective dyads (see also Fig 5D–5I). Note that this is a genuine out-of-sample test, since model parameters previously fitted (see Methods) were held fixed to account for this new data.

Although the model predicted a slightly shorter POR latency for the semitone (m2) dyad than observed (see Discussion), latency predictions match the experimental trend; moreover, the differential response to consonant (P1, M3, P5) and dissonant (m2, TT, m7) dyads found in the MEG data was accurately replicated by the model (latency of P1 and P5 < latency of dissonant dyads: *p* < 10^−7^, *W* > 5050; latency of M3 < latency of m2: *p* = .00002, *W* = 4414; latency of M3 < latency of TT: *p* = .38, *W* = 3688; latency of M3 < latency of m7: *p* = .096, *W* = 3878; one-tailed Wilcoxon rank-sum tests performed over the results of *N* = 60 runs of the model). The full temporal dynamics of the dipole moment predicted by the model is shown in Fig 5D–5I.

Last, we extended the POR latency predictions to all 13 dyads comprising the entire chromatic scale (see Fig 5K), and tested if the differential responses to consonance and dissonance were generalizable to additional dyads. Following Helmholtz [6], we considered an extended set of consonant dyads, including the octave (P8) and the perfect fourth (P4); and an extended set of dissonant dyads, including the major seventh (M7) and the major second (M2). Once again, consonant dyads produced shorter latencies than dissonant dyads (latencies of P1, P4, P5 and P8 < latencies of the extended set of dissonant dyads: *p* <.0003, *W* > 4445; latency of M3 < latency of M2: *p* = .00002, *W* = 4420; latency of M3 < latency of M7: *p* = .75, *W* = 3501; one-tailed Wilcoxon rank-sum tests, *N* = 60). These results, fully in line with our previous findings, suggest that the differential response of our model to consonance and dissonance is a consequence of the harmonic relationships between the periodicities of the two dyad components. These analyses are extended to further families of dyads in S9 Fig, yielding similar results.

The model can also be used to explain how interactions between the components of the dyads influence processing time: consonant dyads consist of tones that share a larger number of lower harmonics than the ones in dissonant dyads. For instance, in the just intonation, the perfect fifth of a given fundamental shares one in every two harmonics with that fundamental, whilst only one in 16 harmonics are shared by a minor second and its fundamental. The proposed mechanism is based on the idea that cortical pitch processing is triggered by the joint activation of, at least, three periodicity detectors characterizing a specific harmonic series. Consonant dyads elicit a dramatically larger signal-to-noise ratio in the periodicity detectors tuned to their common harmonics, resulting in a collaborative effort towards pitch extraction that effectively speeds up processing dynamics (see video S2 Video for an animation depicting the full process).

## Discussion

This work combines new theoretical and experimental findings to elucidate how human auditory cortex processes pitch and consonance/dissonance through similar network mechanisms.

First, we introduced a novel ensemble model designed to reproduce the neuromagnetic fields elicited in alHG during pitch processing. The model was used to understand the POR morphology and the dependence of its peak latency on the perceived pitch, a phenomenon that although robustly observed for over two decades [9], has remained poorly understood. Second, we designed an MEG protocol to investigate whether the POR properties are influenced by the degree of consonance or dissonance, as elicited by different dyads common in Western music. Our results revealed a strong correlation between the POR peak latency and the degree of consonance elicited by each dyad, extending previous EEG results that also reported a modulation of the POR amplitude by consonance and dissonance [2].

Third, we showed that our model (originally designed to explain pitch processing in IRN stimuli with a single pitch) quantitatively replicates the correlation between POR latency and the degree of consonance and dissonance. We provide a mechanistic explanation for the shorter POR latencies in response to consonant dyads as an effect of the harmonic facilitation during pitch processing. Combined, our results indicate that the neural mechanisms accounting for pitch processing show differential responses to consonant and dissonant dyads, showing that the sensation of consonance may be initially elicited as a result of pitch coding in alHG before subsequent cognitive processing.

### The POR latency reflects pitch processing time

A new systematic interpretation of the POR latency can be deduced from the dynamics of the decoder network: the POR might reflect the amount of time that is necessary for the network to robustly stabilize in a state representing a unequivocal pitch (see Fig 2). Although an association between POR latency and processing time has been previously hypothesized in experiments (such as in [9]) and in a phenomenological model [45], a detailed understanding of this mechanism was still lacking. In our model, the latency of the POR coincides with the instant in which the net inhibition at the decoder network overtakes the excitatory activity from the periodicity detectors. From a dynamic systems perspective, this is equivalent to the instant in which the trajectory in the phase-space is unequivocally directed towards the attractor state dominated by the neural ensemble that is characterized by the perceived pitch (see the phase space portrait in S1 Video).

The model only performs a robust perceptual decision concerning stimulus pitch after the cortical system identifies three peaks from the harmonic series of the stimulus period in the representation of the periodicity detectors. This accounts for the relation between the POR latency and the stimulus period [9]. In addition, this also explains why pitch identification is only robust when the stimulus duration exceeds four times the pitch periodicity [9]. Although previous studies had postulated that cortical pitch processing mechanisms must integrate along several period cycles in order to make a perceptual decision [9], a specific mechanism for such an integration has not been proposed to the date.

Moreover, since phase-locked activity is not robustly present above 50–200 Hz in the cortex [15], integration along several repetition cycles is only possible in subcortical areas. The decoder network in our model takes advantage of the input harmonic representations provided by an autocorrelation model that does not require phase-locking to transmit information concerning several repetition cycles [28], and thus provides a parsimonious solution to this problem.

### Effect of consonance and dissonance on cortical processing time

Combined, our results suggest that cortical processing of dissonant dyads is slower than the processing of consonant dyads; i.e., it requires a longer processing time. The model constitutes a physical rationale for this phenomenon: cortical extraction of consonance is based on the accumulation of activity in the columns with preferred periods characterizing the lower harmonics of the target sound; thus, concurrent pitch frequencies sharing common lower harmonics contribute to the build-up of each other’s representation, thereby speeding up the stabilization of the network. Since consonant dyads are characterized by simpler frequency ratios, their components share a larger number of lower harmonics than the components of dissonant dyads, and hence this stabilization is promoted.

Early phenomenological models based on Helmholtz’s roughness theory described dissonance as the beating sensation produced by tones with fundamental periodicities that were not harmonically related [6, 7]. More recent explanations of consonance, based on pitch processing, have linked the regularity of the autocorrelation harmonic patterns elicited by dyads to their evoked consonance and dissonance percepts [1, 4, 19]. Thus, previous phenomenological consonance models have consistently described the degree of consonance as the perceptual correlate of the degree of overlap between the dyad components’ lower harmonics. Our model introduces a potential explanation for the biophysical rationale underlying this description.

Although our modeling results generally show a good fit with the data from the MEG experiment, the model prediction falls around 5 ms short when explaining the POR latency evoked by the minor second dyad. This underestimation might result from the limited number of harmonics considered during the integration step in the decoder network: dissonant dyads, whose components do not share any common harmonic within the first three peaks of their harmonic series, present comparable processing times. More accurate results would occur if an adaptive mechanism adjusted the number of harmonics required to trigger the decoding process according to the degree of peak overlap in the input. This adaptive mechanism would be necessary to explain how humans can differentiate dyads that differ in a quarter of a semitone.

Our study did not assess whether the general (yet not universal [3, 5]) association between consonance and pleasantness might be a consequence of the differential responses of the decoder network to consonant and dissonant dyads. Future work should investigate whether this link could be better explained by processes at higher levels of the auditory hierarchy that might be more sensitive to cultural and background modulations.

### Comparison with previous experimental results

Our neuromagnetic findings concerning the POR morphology in response to consonant and dissonant dyads resemble and extend recent EEG data reported by Bidelman and Grall [2], and by Proverbio et al. [48]. Specifically, Bidelman and Grall [2] applied EEG in a smaller sample (*N* = 9) of musically trained listeners and revealed a close relation between their subject’s consonance/dissonance ratings and the morphology of the POR that was elicited by the respective dyads in alHG. In their study, the POR latency difference between consonant and dissonant dyads (cf. their Fig 4b) appears to have a non-significant (*p* = .22) effect size, smaller than the results that were obtained in our study by means of MEG.

One reason for this might be that Bidelman and Grall [2] applied shorter IRN stimuli with a higher number of iterations, resulting in an increased saliency of the pitch percept; moreover, they employed a dichotic stimulation paradigm in which each ear was presented with only one dyad component, whereas in our experiment sounds were delivered diotically to the listeners. Since our model does not predict an effect of the number of iterations of the IRNs on latency (predictions for dyads of 32 iterations IRNs are shown in S9D Fig), we speculate that the diotic presentation of the dyads is responsible for the stronger effect shown in our data. This hypothesis cannot be explored by our current model because it does not consider binaural integration. The modeling of this process could be informed by the divergence between Bidelman and Grall’s and the present results in future work.

In line with results from previous experiments (e.g., [9, 11, 30, 32, 49]), our findings are consistent with the notion that lateral HG acts as a cortical pitch center. Many of these earlier studies employed IRN stimuli; however, a number of fMRI experiments (e.g., [50, 51]) have argued that the activity observed in HG might be confounded by slow fluctuations in the IRN spectrum. Indeed, pitch-sensitive cell ensembles seem to overlap, within HG, with other neural populations that are more sensitive to other (e.g., spectral) sound features [52, 53]. However, this does not speak against the existence of a pitch-specialized subregion in HG since overlapping neuron ensembles are difficult to disentangle by means of fMRI [54]. Thus, based on the relatively homogeneous pattern of results in the current study and in experiments using different stimuli and neuroimaging methods [2, 18, 31, 35], the existence of a pitch center in HG might be viewed as highly probable.

### Relation to pitch perception models

Numerous phenomenological models have been designed to predict pitch for a wide range of complex sounds (e.g., [24, 27, 28, 45, 46, 55], see [47] for a review). These models can account for a variety of perceptual phenomena [24]. The weaker pitch of frequency-transposed harmonic complex sounds [56], for example, was explained using nonlinear filters to simulate the compression taking place in the basilar membrane [24]. Although the present work constitutes a first effort towards a mechanistic model of early pitch and consonance processing, future efforts should focus on broadening the model scope to pitch phenomena not addressed in the current model implementation.

The correlation between pitch and cortical AEFs has been qualitatively studied in the Auditory Image Model’s *buffer* [46] and its derivative [57], and quantitatively in the derivative of the model output in [45] and [10]. However, these models did not provide a mechanistic explanation of the processes underlying the generation of the POR or its latency dependence with pitch.

Other models, designed to explain the biophysical mechanisms of pitch perception, focused primarily on subcortical processing. Two of these models describe how neurons, mainly in subcortical nuclei, might process periodicities from the auditory nerve activity: Meddis and O’Mard’s model [23] proposes a biophysical implementation of the summary autocorrelation function [27, 28], based on the joint action of chopper neurons in the cochlear nucleus and coincidence detectors in the inferior colliculus. More recently, Huang and Rinzel [58] described a neural implementation of an array of coincidence detectors able to detect periodicities by comparing neural activity across different cochlear channels. Despite their mechanistic differences, both models present an output comparable to that of the autocorrelation function [58]. The model presented here is downstream with respect to Meddis’ and Huang’s models because it focuses on explaining how pitch decisions based on the later subcortical representation are made in alHG.

### Biological plausibility

In our model, pitch processing is mediated by a connectivity pattern among interacting columns specialized in characteristic periods. Similar connectivity patterns were found in mice AC, stemming from L4 and targeting L6 neurons [36], and in the cat AC in earlier studies [37]. Neurons that respond selectively to harmonically related input have been recently identified in the core region of the marmoset’s auditory cortex [13].

Inhibitory and facilitatory interactions between neurons encoding harmonically related frequencies are often reported in the mammal auditory cortex (see [59] for a review). Specifically, intracranial recordings in marmoset AC revealed that activation elicited by a given tone resulted in the facilitation of neurons encoding higher harmonics, and in the suppression of neurons encoding lower harmonics [60], in line with the decoder network mechanisms of our model. Harmonic co-activation has also been shown in human AC [61].

In a more speculative vein, we suggest that this connectivity pattern might result from spike-timing-dependent plasticity (STDP) operating over cortical neurons receiving the upstream outputs of periodicity detectors. To illustrate this, let us consider the processing of a sound of fundamental period *T*. After tone onset, the first periodicity detector responding to the sound provides input to the upstream excitatory ensemble encoding *T*, which subsequently activates its inhibitory counterpart. Assuming an initial all-to-all connectivity, this inhibitory drive propagates in the network and provides concurrent input to neurons receiving excitatory drive from periodicity detectors 2*T*, 3*T*, and so forth. Input synchrony would result in a stronger connectivity change though STDP in these harmonically related ensembles, whilst the uncorrelated asynchronous inputs to the remaining ensembles would result in a net decreased connectivity weight. A similar STDP mechanism for spectral pitch integration was proposed earlier in [62].

The *decoding* strategy or our model is based on the well-known winner-takes-it-all architecture [29, 63, 64]: excitatory populations in the decoding network compete with each other, while the inhibitory ensemble arbitrating this competition is the one in the column that is sensitive to the fundamental period (Fig 1). In this way, multiple fundamentals can be simultaneously decoded (Fig 5). Moreover, akin to recent models of sensory integration [29], once a fundamental period is represented in the decoder network, the activity of the winner column is reinforced by the sustainer network (rather than the pitch being repeatedly decoded). This ensures stability until a significant change in the subcortical input triggers a new decoding process (see S2 Fig).

This *sustaining* strategy is also related to predictive coding-related strategies [45, 65, 66], where top-down efferent convey expectations about the input, whereas bottom-up afferents convey prediction errors [65]. Additional top-down expectations could coexist at higher cognitive levels based on, for example, prior knowledge, experience, or focused attention. Such biases could modulate the sustainer network by increasing the baseline activity of the inhibitory ensembles that characterize the target pitch values, thereby facilitating pitch processing in the decoder network.

To summarize, in this study we proposed a model specifically designed to understand the neural mechanisms of cortical pitch processing at a mesoscopic scale. We introduce a possible mechanistic link between the latency of the POR component in the N100 deflection and the processing time required for the system to achieve convergence, explaining the classical result that tones with a lower pitch elicit PORs with longer latencies. More intriguingly, our modeling and experimental results indicate that processing time varies with the degree of consonance in dyads, suggesting that the sensation of consonance and dissonance might start early in auditory cortex, prior to higher-order processing.

## Methods

### Experimentation

#### Participants

Thirty-seven normal-hearing adults (22 female, 2 left-handed; mean age: 29.1 ± 8.3 years) participated in the experiment. The size of the study sample was chosen in an effort to reliably segregate the neuromagnetic responses to highly consonant and highly dissonant dyads, thereby exceeding the sample sizes reported in previous studies [21, 48]. None of the subjects reported any history of central or peripheral hearing impairments or any neurological or psychiatric disorders. The study and the experimental procedures were approved by the ethics committee of the Medical Faculty of the University of Heidelberg, and were conducted with written informed consent of each listener.

#### Stimuli

All stimuli were generated on-line using MATLAB 7.1 (The MathWorks, Inc., USA) and a sampling rate of 48000 Hz. The basic stimulus was a 750 ms long IRN segment, bandpass filtered at 125–2000 Hz, with eight iterations and gain for the delay-and-add filter *g*_*f*_ = 1:

$$\begin{array}{c} s\left(t\right)=\sum_{n=0}^{\#\text{its}-1}g_{f}^{n}\;(s_{0}\left(t-n\;T_{0}\right)+s_{0}\left(t-n\;T_{1}\right)), \end{array}$$ (1)

where *s*_0_(*t*) is the sound waveform of a continuous white noise. The delays of the IRNs *T*_0_ and *T*_1_ were varied between experimental conditions in order to build three consonant and three dissonant musical intervals, as classified by Western music theory. The delay of the lower note was always *T*_0_ = 6.25 ms, corresponding to a pitch of 160 Hz; the delay of the upper note *T*_1_ = 2^−(*f* ratio)^ *T*_0_ was adjusted accordingly to form either a consonant dyad (unison, P1; perfect fifth, P5; major third, M3) or a dissonant dyad (tritone, TT; minor seventh, m7; minor second, m2). Table 1 presents an overview of the six experimental conditions.

**Table 1:** Overview of the experimental conditions. Dyads are listed in descending consonance order, and are categorized as perfect consonant (PC), imperfect consonant (IC) or dissonant (D) according to Western music theory and empirical results [68].

musical interval	*f* ratio	*f* (rounded, Hz)	cons. percept
**unison** (P1)	1:1	160 / 160	PC
**perfect fifth** (P5)	3:2	240 / 160	PC
**major third** (M3)	5:4	200 / 160	IC
**tritone** (TT)	45:32	225 / 160	D
**minor seventh** (m7)	16:9	284 / 160	D
**minor second** (m2)	16:15	171 / 160	D

In order to separate the dyad-specific neuromagnetic responses from the cortical activity associated with the onset of sound energy [67], each IRN dyad was preceded by a 750 ms long, energy-balanced noise segment (bandpass filtered from 125 Hz to 2000 Hz). There were 10 ms Hanning windows at stimulus onset and offset. Moreover, between the first (noise) and the second (IRN) segment of a stimulus, signals were cross-faded for a duration of 10 ms to avoid discontinuous waveforms. The overall stimulation level was set to 80 dB SPL.

#### Data acquisition and processing

Gradients of the magnetic field were acquired with a Neuromag-122 whole-head MEG system (Elekta Neuromag Oy, Helsinki, Finland) inside a magnetically shielded room (IMEDCO, Hägendorf, Switzerland). Raw data were low-pass filtered at 330 Hz and acquired at a sampling rate of 1000 Hz. Prior to the recordings, the nasion, two pre-auricular points and 32 surface points were measured as anatomical landmarks, individually for each participant, using a Polhemus 3D-Space Isotrack2 systems. In order to keep vigilance stable, participants watched a silent movie of their own choice during data acquisition, and they were asked to direct their attention to the movie and ignore the sounds in the earphones. IRN dyads were delivered to the subjects via Etymotic Research (ER-3) earphones with 97 cm plastic tubes and foam earpieces. Sounds were presented using a 24-bit sound card (RME ADI 8DS AD/DA interface), an attenuator (Tucker-Davis Technologies PA-5), and a headphone buffer (Tucker-Davis Technologies HB-7). 250 sweeps per stimulus condition were played during the MEG recording, diotically and in pseudo-randomized order. The inter-stimulus interval was 1000 ms. The total duration of the measurement was 62 minutes.

#### Data analysis

Data were analyzed off-line using the BESA 5.2 software package (BESA GmbH, Germany) with a spherical head model and a homogeneous volume conductor. After visual inspection of the raw data, noisy channels and sweeps with amplitudes greater than 8000 fT/cm or gradients exceeding 800 fT/cm ms were excluded from further analyses. About 235 sweeps per subject and condition remained after artifact rejection; they were averaged, trigger-synchronously, in the epoch from 500 ms before to 3000 ms after stimulus onset. The baseline was defined as the average level in the interval of -100 ms to 0 ms, relative to stimulus onset.

After pre-processing, we applied spatio-temporal source models [69] in BESA, to study the POR component in response to the second stimulus segment; i.e., at the transition from noise to IRN dyads. In this source localization approach, the intracortical sources of the activity observed at the scalp are modeled as equivalent current dipoles, and their spatial position and orientation is varied iteratively until a maximum amount of variance is explained in the scalp data. The source model includes both, the spatial information for each dipole and its physiological activity across time (source waveform). We calculated source models with one dipole per hemisphere for the POR component in the second stimulus segment. Dipole fits were based on pooled conditions [P1+P5+M3+TT+m7+m2]. The fitting interval covered about 30 ms around its peak, and MEG data were zero-phase filtered 1–20 Hz.

Individual fits at the AEF components were successful for 36 subjects. In ten participants we included a symmetry constraint in the model to stabilize the individual dipole fits. One participant failed to show stable fits in the dipole model and was excluded from subsequent analyses. Aside from symmetry, no further constraints were made concerning the orientation and location of the dipoles. The average maximum of explained variance within the fitting window was 64.1% (SD: 18.9) for the POR dipole model. After fitting, this dipole model was used as spatio-temporal filter; i.e., the source waveforms corresponding to the model were extracted separately for each condition and each subject. Finally, the source waveforms were exported from BESA to MATLAB for statistical analysis.

The statistical evaluation of the MEG source waveforms was conducted using the bootstrap method. Here, the distribution of a test statistic is approximated by repeated random drawing, with replacement, from the original dataset; based on the resulting bootstrap distribution, confidence intervals can then be derived for that test statistic. Contrary to most standard techniques, the bootstrap method is well-suited for neurophysiological data where peaks cannot be clearly identified for each participant in every condition. Prior to statistical analyses, each source waveform of the POR model was adjusted to the baseline calculated as the average of the last 100 ms before the transition.

Processed MEG data are publicly available in the Open Science Framework repository: http://osf.io/chqvf.

### Modeling

#### Peripheral model and periodicity detectors

Neural activity in the auditory nerve was simulated using a recent biophysically realistic model of the auditory periphery [26]. Peripheral model parameters were chosen as in [23], considering 40 cochlear channels with center frequencies between 125 Hz and 10 kHz.

Periodicity detectors were modeled according to the summarized autocorrelation function (SACF) of the auditory nerve activity [23, 24, 28]. This idealized model yields a harmonic neural representation of pitch-related information (see Fig 1E). The SACF was chosen for its low computational cost, but more detailed biophysical models produce similar representations (e.g., [23, 58]).

The SACF used here follows the same formulation as the first stage in the cascade autocorrelation model [24]:

$$\begin{array}{c} \tau_{n}^{\text{SACF}}{\dot{A}}_{n}\left(t\right)=-A_{n}\left(t\right)+\sum_{m}p{\left(t\right)}_{m}\;p\left(t-\delta t_{m}\right), \end{array}$$ (2)

where *p*(*t*)_*m*_ is the instantaneous spiking probability of the cochlear channel *m*, and $\tau_{n}^{\text{SACF}}$ are the SACF integration time-constants [24, 38]. The *n*^th^ component *A*_*n*_(*t*) of the SACF as described in Eq (2) represents a measure of regularity in the auditory nerve activity with respect to a fixed period *δt*_*n*_. The model considers *N* = 250 of such periods uniformly spaced between *δt*_1_ = 0.5 ms, a conservative estimation of the phase-locking limit of the auditory nerve [70], up to the lower limit of melodic pitch, *δt*_*N*_ = 30 ms [71].

The output is further regularized through a procedure $A_{n}(t)\to {\hat{A}}_{n}(t)$ that reduces the dependence of the SACF with stimulus intensity level and minimizes signal-to-noise variations in sounds with the same pitch but different timbre. The regularization procedure is based on neuronal normalization principles [72] (see S3 Text).

#### Ensemble dynamics

Neural ensembles follow mean-field dynamics adapted from [64], characterized by their instantaneous firing rates $H_{n}^{e}(t)$ (excitatory) and $H_{n}^{i}(t)$ (inhibitory) at each cortical column *n*:

$$\begin{array}{c} \tau^{\text{pop}}\;{\dot{H}}_{n}^{e,i}\left(t\right)=-H_{n}^{e,i}\left(t\right)+\phi^{e,i}\left(I_{n}^{e,i}\left(t\right)\right). \end{array}$$ (3)

where *τ*^pop^ is the population time constant (see Eq (5)) and $\phi^{e,i}(I_{n}^{e,i}(t))$ are the transfer functions [64]:

$$\begin{array}{c} \phi^{e,i}\left(I\right)=\frac{a^{e,i}I-b^{e,i}}{1-e^{-d^{e,i}\left(a^{e,i}I-b^{e,i}\right)}}. \end{array}$$ (4)

Realistic parameters of excitatory and inhibitory transfer functions (*a*^*e*^, *b*^*e*^ and *d*^*e*^ for the excitatory; *a*^*i*^, *b*^*i*^ and *d*^*i*^ for the inhibitory) were taken from the literature [64, 73]. The total synaptic inputs $I_{n}^{e}(t)$ and $I_{n}^{i}(t)$ are defined below. Numerical simulations were performed using the Euler’s method with a time step Δ*t* = 1 ms.

The dynamics of excitatory and inhibitory ensembles of the *decoder* and *sustainer* networks follow the same formulation. In order to differentiate between the two networks, we use $H_{n}^{e,i}(t)$ and $I_{n}^{e,i}(t)$ to characterize populations and synaptic inputs of the decoder layer and ${\hat{H}}_{n}^{e,i}(t)$ and ${\hat{I}}_{n}^{e,i}(t)$ for the populations and synaptic inputs of the sustainer layer. Population effective time constants *τ*^pop^ are adaptive and depend on the activity of the population [74]:

$$\begin{array}{c} \tau^{\text{pop}}\left(H\left(t\right)\right)=\tau_{0}^{\text{pop}}\;\Delta_{T}\frac{\phi^{\prime }\left(I\left(t\right)\right)}{H(t)}, \end{array}$$ (5)

where Δ_*T*_ = 1mV is the sharpness of the action potential initiation [74] and

$$\begin{array}{c} \phi^{\prime }\left(I\right)=\partial_{x}{\phi \left(x\right)|}_{x=I}=a\phi \left(I\right)(\frac{1}{aI-b}+\frac{d}{1-e^{-d(aI-b)}}). \end{array}$$

#### Synaptic dynamics

Ensemble connectivity is mediated through realistic AMPA, NMDA and GABA_A_ synapses [63, 64, 73]. Synaptic dynamics were modelled according to Brunel’s formulation [73]:

$$\begin{array}{c} {\dot{S}}_{n}^{\left\{\text{AMPA,}\;\text{GABA}\right\}}\left(t\right)=-\frac{S_{n}^{\left\{\text{AMPA,}\;\text{GABA}\right\}}\left(t\right)}{\tau_{\left\{\text{AMPA,}\;\text{GABA}\right\}}}+H_{n}^{\left\{e,i\right\}}\left(t\right)+\xi \end{array}$$

$$\begin{array}{c} {\dot{S}}_{n}^{\text{NMDA}}\left(t\right)=-\frac{S_{n}^{\text{NMDA}}\left(t\right)}{\tau_{\text{NMDA}}}+\gamma (1-S_{\text{NMDA}}\left(t\right))H_{n}^{e}\left(t\right)+\xi . \end{array}$$

The NMDA time constant was set to *τ*_NMDA_ = 30 ms; GABA and AMPA time constants *τ*_GABA_ = 2 ms and *τ*_AMPA_ = 5 ms, and the coupling parameter *γ* = 0.641, were all taken from the literature [64, 73]. The last terms in the equations *ξ* = *σν*_*n*_(*t*) introduce noise in the synaptic gating variables through Wiener processes *ν*_*n*_(*t*) with mean zero and variance *σ* = 0.0007 nA [64] that are independently sampled for each variable and time instant. Gating variables of the sustainer and decoder layers ${\hat{S}}_{n}^{\text{NMDA, AMPA, GABA}}(t)$, ${\hat{H}}_{n}^{e,i}(t)$ follow similar dynamics.

#### Synaptic inputs

The total synaptic inputs to populations $I_{n}^{i,e}(t)$ and ${\hat{I}}_{n}^{i,e}(t)$ in Eq (3) consist of three different contributions: internal input *I*_int_, accounting for inputs from populations within the same network, external input *I*_ext_, exerted by sources from other networks, and a constant input drive *I*_0_: $I_{n}^{i,e}(t)=I_{n,\text{int}}^{i,e}(t)+I_{n,\text{ext}}^{i,e}(t)+I_{n,0}^{i,e}(t)$ and ${\hat{I}}_{n}^{i,e}(t)={\hat{I}}_{n,\text{int}}^{i,e}(t)+{\hat{I}}_{n,\text{ext}}^{i,e}(t)+{\hat{I}}_{n,0}^{i,e}(t)$.

*Internal input*. Connectivity weights between any two ensembles in the decoder network are provided by the matrices *C*^*ee*^, *C*^*ei*^, *C*^*ie*^, *C*^*ii*^. *C*^*ei*^ and *C*^*ie*^ present a harmonic structure inspired in connectivity patterns reported in the mammal auditory cortex (see Discussion); these matrices are plotted in Fig 1D. *C*^*ee*^ is the identity matrix, and *C*^*ii*^ has a similar diagonal structure: $C_{\alpha \beta }^{ii}=(1-c_{0}^{ie})\delta_{\alpha \beta }+c_{0}^{ie}$, where $c_{0}^{ie}$ is the baseline inhibitory weight $c_{0}^{ie}=0.1$ and *δ*_*αβ*_ is the Kronecker delta. The internal inputs to the decoder *I*_int_(*t*) are defined as follows:

$$\begin{array}{ccc} I_{n,\text{int}}^{\alpha }\left(t\right) & = & \sum_{k}C_{nk}^{e\alpha }(J_{\text{NMDA}}^{e\alpha }\;S_{k}^{\text{NMDA}}\left(t\right)+J_{\text{AMPA}}^{e\alpha }\;S_{k}^{\text{AMPA}}\left(t\right))\\ & & -\sum_{k}C_{nk}^{i\alpha }J_{\text{GABA}}^{i\alpha }\;S_{k}^{\text{GABA}}\left(t\right),\;\alpha =e,i \end{array}$$ (6)

Ensembles in the sustainer network only communicate internally with populations within the same block:

$$\begin{array}{ccc} {\hat{I}}_{n,\text{int}}^{e}\left(t\right) & = & {\hat{J}}_{\text{NMDA}}^{ee}\;{\hat{S}}_{n}^{\text{NMDA}}\left(t\right)+{\hat{J}}_{\text{AMPA}}^{ee}\;{\hat{S}}_{n}^{\text{AMPA}}\left(t\right) \end{array}$$ (7)

$$\begin{array}{ccc} & & -{\hat{J}}_{\text{GABA}}^{ie}\;{\hat{S}}_{n}^{\text{GABA}}\left(t\right) \end{array}$$ (8)

$$\begin{array}{ccc} {\hat{I}}_{n,\text{int}}^{i}\left(t\right) & = & {\hat{J}}_{\text{AMPA}}^{ei}\;{\hat{S}}_{n}^{\text{AMPA}}\left(t\right) \end{array}$$ (9)

Conductivities *J*_NMDA,AMPA,GABA_ and ${\hat{J}}_{\text{NMDA},\text{AMPA},\text{GABA}}$ (see Table 2) were initialized to typical values in the literature *J* ≃ 0.15 nA [64], and fine-tuned within a range of realistic values to ensure the convergence of the ensembles activity for single-pitch iterated rippled noises. The excitatory-to-inhibitory conductivity $J_{\text{AMPA}}^{ei}$ was further adjusted such that three harmonics were necessary to perform a perceptual decision. This enables the model to replicate the dependence of the POR with pitch in two reference IRNs with periods *T* = 2 ms and *T* = 8 ms. Model’s final parameters are listed in Table 2, and are held fixed for the rest of the stimuli analysed in this study.

**Table 2:** Values for the parameters used in the cortical model. The last column specifies the source of the parameter value; entries without a reference were tuned within the range of realistic values. Time constants for synaptic dynamics were taken from the original formulation of the models referenced in this work. All values were grounded in empirical data; e.g., $\tau_{{\text{GABA}}_{\text{A}}}^{decay}≃2-8$ ms [75], $\tau_{\text{AMPA}}^{decay}=(2\pm 0.8)$ ms [76], *τ*^pop^ = (11.9±6.5) ms in fast spiking cortical neurons [77]. Similarly, in synapses targeting inhibitory neurons, $\tau_{\text{NMDA}}^{decay}\in [11.6,27.1]\;$ ms [78].

par	value	description	source
$J_{\text{AMPA}}^{th}$	2.7 nA	conductivity of the subcortical input	-
$J_{\text{NMDA}}^{e}$	0.45 nA	top-down (sustainer to decoder) conductivity	-
${\hat{J}}_{\text{GABA}}^{a}$	0.45 nA	bottom-up (dec to sust) inh conductivity	-
${\hat{J}}_{\text{AMPA}}^{a}$	0.35 nA	bottom-up (dec to sust) exc conductivity	-
$J_{\text{NMDA}}^{ee}$	0.14 nA	decoder’s exc-exc NMDA conductivity	-
$J_{\text{AMPA}}^{ee}$	0.00099 nA	decoder’s exc-exc AMPA conductivity	[64]
$J_{\text{NMDA}}^{ei}$	0.17 nA	decoder’s exc-inh NMDA conductivity	-
$J_{\text{AMPA}}^{ei}$	0.000065 nA	decoder’s exc-inh AMPA conductivity	[64]
$J_{\text{GABA}}^{ie}$	0.53 nA	decoder’s inh-exc conductivity	-
$J_{\text{GABA}}^{ii}$	0.11 nA	decoder’s inh-inh conductivity	-
${\hat{J}}_{\text{NMDA}}^{ee}$	0.25 nA	sustainer’s exc-exc NMDA conductivity	-
${\hat{J}}_{\text{AMPA}}^{ee}$	0.00099 nA	sustainer’s exc-exc AMPA conductivity	[64]
${\hat{J}}_{\text{AMPA}}^{ei}$	0.00099 nA	sustainer’s exc-inh AMPA conductivity	[64]
${\hat{J}}_{\text{GABA}}^{ie}$	0.80 nA	sustainer’s inh-exc conductivity	-
$c_{0}^{ie}$	0.1	ratio between global and specific inhibition	-
*γ*	0.641	coupling parameter of NMDA synaptic gating	[73]
*a*^*e*^	310 (VnC)^−1^	transfer function parameter for exc ensembles	[64]
*b*^*e*^	125 Hz	transfer function parameter for exc ensembles	[64]
*d*^*e*^	0.16 s	transfer function parameter for exc ensembles	[64]
*a*^*i*^	615 (VnC)^−1^	transfer function parameter for inh ensembles	[64]
*b*^*i*^	177 Hz	transfer function parameter for inh ensembles	[64]
*d*^*i*^	0.087 s	transfer function parameter for inh ensembles	[64]
$I_{0}^{e}$	0.315 nA	decoder’s baseline excitatoy input current	-
$I_{0}^{i}$	0.15 nA	decoder’s baseline inhibitory input current	-
${\hat{I}}_{0}^{e}$	0.26 nA	sustainer’s baseline excitatoy input current	-
${\hat{I}}_{0}^{i}$	0.18 nA	sustainer’s baseline inhibitory input current	-
*τ*_AMPA_	2 ms	time constant of the AMPA decay	[73]
*τ*_GABA_	5 ms	time constant of the GABA decay	[73]
*τ*_NMDA_	30 ms	time constant of the NMDA decay	-
*τ*^pop^	10 ms	membrane time constant	[74]
Δ_*T*_	1 mV	sharpmness fo the action potential initiation	[74]
*σ*	0.0007 nA	variance of the synaptic noise	[64]

*External input*. Excitatory ensembles in the decoder network receive bottom-up input ${\hat{A}}_{n}(t)$ via AMPA-driven synapses, in line with previous studies on perceptual integration [64]:

$$\begin{array}{c} I_{n,\text{ext}}^{e}\left(t\right)=J_{\text{AMPA}}^{th}\;S_{n}^{th,\text{AMPA}}\left(t\right). \end{array}$$

The conductivity $J_{\text{AMPA}}^{th}$ was adjusted to ensure a smooth and robust propagation of the activity in the periodicity detectors to the decoder’s excitatory populations. The corresponding gating variables $S_{n}^{th,\text{AMPA}}(t)$ follow AMPA-like dynamics:

$$\begin{array}{c} {\dot{S}}_{n}^{th,\text{AMPA}}\left(t\right)=-\frac{S_{n}^{th,\text{AMPA}}\left(t\right)}{\tau_{\text{AMPA}}}+A_{n}\left(t\right). \end{array}$$ (10)

Inhibitory ensembles in the decoder receive efferent external input from the sustainer network. Top-down excitatory processes in cortex are typically dominated by NMDA dynamics [65]; thus, efferent AMPA synapses were not considered:

$$\begin{array}{c} I_{n,\text{ext}}^{i}\left(t\right)=J_{\text{NMDA}}^{e}\;{\hat{S}}_{n}^{th,\text{NMDA}}\left(t\right). \end{array}$$

The efferent conductivity $J_{\text{NMDA}}^{e}$ (Table 2) was tuned to facilitate the timely top-down enhancement of inhibitory ensembles at the decoder (see details *Dynamics of the decoder network* in Results).

Sustainer’s external inputs originate from the decoder network, driven by inhibitory GABAergic ${\hat{I}}_{n,\text{ext}}^{i}(t)={\hat{J}}_{\text{GABA}}^{a}\;S_{n}^{\text{GABA}}(t)$ and excitatory AMPAergic ${\hat{I}}_{n,\text{ext}}^{e}(t)={\hat{J}}_{\text{AMPA}}^{a}\;S_{n}^{\text{AMPA}}(t)$ synapses [64, 65]. Afferent conductivities ${\hat{J}}_{\text{AMPA, GABA}}^{a}$ (Table 2) were set to make the sustainer both sensitive to decoded decisions, yet robust to spurious activations.

*Constant input drive*. Constant inputs to the decoder $I_{n,0}^{e}(t)=I_{0}^{e}$ and $I_{n,0}^{i}(t)=I_{0}^{i}$ (Table 2) were selected to enable the system to be reactive to external input, yet silent in absence of a significant input. An additional constant drive $I_{0}^{\text{sus}}=0.24\;$ nA was applied to the populations at the sustainer (see *Dynamics of the sustainer network* in Results).

#### Derivation of the evoked fields

Assuming that all microcolumns within each of the two cortical networks present similar orientations, the total dipolar moment representing the neuromagnetic field elicited by each network is monotonically related to the collective excitatory activity in the network [40]: $m(t)=\sum_{n}H_{n}^{e}(t+\Delta t_{\text{subcort}})$.

The subcortical processing time Δ*t* accounts for the time elapsed from tone onset until the signal first arrives to the decoder network in cortex. This delay reflects propagation time and subcortical processes such as the regularization of the output of the periodicity detectors. The subcortical delay was fixed to Δ*t* = 50 ms such that the model predicts the POR latency for a reference IRN of a 1/8 Hz pitch. We used a larger Δ*t*^dyads^ = 75 ms in dyads to compensate for a systematic 25 ms delay observed between the model predictions and experimental observations for dyads. We speculate that this difference is due to the different rescaling factors used for the regularized SACF in single tones and dyads (see details in S3 Text).

The implementation of the model used to produce all the results and a script reproducing the figures are publicly available in a Github repository: http://github.com/qtabs/moch.

## Supporting information

S1 Video Model dynamics during the processing of iterated rippled noise. a) Instantaneous firing rate of the periodicity detectors (yellow) and the ensembles in the decoder (excitatory blue, inhibitory red). b) Instantaneous firing rate of the ensembles in the sustainer (excitatory blue, inhibitory red). c) Two-dimensional projection of the state variables of the decoder during pitch processing; projection axes were chosen as the two first principal components (PCA) of the decoder’s variables (i.e. the firing rates of the neural ensembles). Each dot represents the state of the system at a given instant *t* with a step size of Δ*t* = 1 ms. Colors were used to characterize the different stages of the model dynamics: open blue circles represent the absence of input (points are too close to each other to be distinguished); red dots represent states within the time window spanning from the stimulus onset to the convergence of the model to a specific pitch value (at about 175 ms after sound onset); yellow dots represent states within temporal windows spanning from the convergence of the system to the tone offset; purple dots show states in the time window corresponding to the the *relaxation dynamics*, spanning form the offset of the tone up to 350 ms after sound offset. d) Excitatory and inhibitory firing rate of the column characterizing the extracted pitch in the sustainer network. Note that the relaxation dynamics of the sustainer, corresponding to the trajectory of the system after offset (purple points), is much slower than the relaxation dynamics of the decoder (resembling the characteristic of the sustained field offset delay [67]). e) Aggregated excitatory activity in the decoder (blue) and the sustainer (red), monotonically related to the equivalent dipole moment of the elicited fields in each of the two networks. Stimulus parameters were chosen according to Krumbholz et al. [9]; i.e., same as in Fig 2 in the main text. Stimulus pitch was set to *f* = 200 Hz (*T* = 5 ms).
(MP4)

S2 Video Model dynamics during the processing of dyads. a)–b) Instantaneous firing rate of the periodicity detectors (yellow), the ensembles in the decoder (excitatory blue, inhibitory red), and the excitatory ensemble in the sustainer (purple) for two IRN dyads: a minor second (a) and a perfect fifth (b). c)–d) Two-dimensional projection of the state variables of the decoder during pitch processing of a minor second (c) and a perfect fifth (d) dyad; see caption of S1 Video for more details. e) Aggregated excitatory activity in the decoder, monotonically related to the predicted elicited field in the generator of the POR, for each of the two dyads: the minor second (blue) and the perfect fifth (red). Note that the system converges earlier for the consonant dyad (the minor fifth), eliciting an earlier POR. Stimulus parameters were chosen as in Figs 4 and 5 in the main text.
(MP4)

S1 FigAttractor dynamics underlying pitch processing.a) Two-dimensional projection of the state variables $\vec{x}$ during pitch processing using principal components analysis (PCA; see caption in S1 Video for details). The trajectory in the reduced space reveals key aspects of the onset and relaxation dynamics; the transition from ${\vec{x}}_{0}$ to ${\vec{x}}_{1}$ characterizes the POR. b) View of the two dimensions of the subsystem characterizing the decoded pitch *n* in the sustainer network (see section S1.2). Note that the relaxation dynamics of the sustainer network, corresponding to the transition from ${\hat{\vec{x}}}_{1}^{n}$ to ${\hat{\vec{x}}}_{0}^{n}$, are much slower than the relaxation dynamics of the decoder network; resembling the sustained field offset delay [67]. See also the caption in S1 Video.(TIF)

S2 FigSystem’s reaction to pitch changes.a) Response to pitch changes (see caption in Fig 2 in the Main Text for details). b)–c) Representation of the attractor dynamics of the model under pitch changes. Two colours were added to represent the new states in the system’s evolution: purple now represents the dynamics from the second stimulus onset to the new state of convergence, defined here as the state achieved 135 ms after the onset; green represents states between convergence and the second stimulus offset; and light blue represent the states during the relaxation dynamics after offset. The remaining colours are kept as in S1 Fig and S1 Video. Note that the transition from ${\vec{x}}_{1}$ to ${\vec{x}}_{2}$ elicits a new, second POR corresponding to the second stimulus. Stimuli were IRNs with the same specifications as in [9]; first tone had a fundamental frequency *f*_0_ = 200 Hz, second tone was two semitones higher than the first note, with *f*_0_ = 225 Hz. The pitch transition occurs 350 ms after the onset of the first tone (see arrow in the figure).(TIF)

S3 Fig Predicted latencies for additional families of IRNs. Except for the number of iterations of the IRNs and the number of runs used to obtain the results (in this case, *N* = 10) simulation parameters were the same as in Fig 3a; error bars are standard deviations. Although experimental data is not available for these stimuli, results faithfully replicate the trends reported in Fig 3a.
(TIF)

S4 Fig Predicted latencies for pure tones. a) Simulated N100 latency values (black error bars) and N100 latency observations (blue error bars); the two experimental curves correspond to latency values observed in the right and left hemispheres. Predictions were averaged along *N* = 10 runs of the model; error bars are standard errors. Experimental data was taken from Roberts et al. [39], Fig 2.
(TIF)

S5 Fig Model representation of pitch for pure tones. Averaged model responses to pure tones at different stages of the model: (a) periodicity detectors, (b/c) excitatory/inhibitory ensembles in the decoder, (d/e) excitatory/inhibitory ensembles in the sustainer. The decay of the responses under *f* ∼ 125Hz (or *T* ∼ 8 ms) is due to the lower-frequency limit of the peripheral model [25]. The Figure was produced using the same methodology as in Fig 3d–3h (see Main Text for details).
(TIF)

S6 Fig Model representation of pitch for click trains. Click trains (generated as a train of Dirac deltas) elicit the same pitch sensation as a sine wave with period *T* equal to the interclick interval [42]. Colormaps show the averaged responses click trains at different stages of the model: (a) periodicity detectors, (b/c) excitatory/inhibitory ensembles in the decoder, (d/e) excitatory/inhibitory ensembles in the sustainer. Results are fully consistent with experimental observations [42].
(TIF)

S7 Fig Model representation of pitch for harmonic complex tones. HCTs elicit the same pitch percept as a sine wave with the frequency of the fundamental of the complex, even if the fundamental itself is not comprised in the complex (known as the *virtual pitch* [41]). The figure shows the responses of the model for: a) HCTs formed by the fundamental and the first 5 higher harmonics; b) HCTs with a missing fundamental (comprising only by the first four higher harmonics); c) HCTs with a missing fundamental comprising harmonics that are not independently resolved in the cochlea (tones were generated as harmonic complexes with harmonics 1 to 50, bandpass filtered between 3.2 kHz and 5 kHz). Note that, since the model uses several peaks of the harmonic series to extract the pitch value from the representation in the periodicity detectors, the perceptual range of the model is limited to periods *T* < 15 ms. Averaged responses in the sustainer populations are precisely correlated with the responses in the inhibitory ensembles in the decoder (omitted here for simplicity).
(TIF)

S8 Fig Model representation of pitch for other classes of iterated rippled noises. The figure shows the perceptual responses for additional classes of iterated rippled noises (IRN) with different parametrisations (see also Fig 3 in the Main Text): a) IRN with 32 iterations and no filtering; b) IRN with 4 iterations and no filtering; c) IRN with 8 iterations, bandpass filtered between 125 Hz and 2 kHz (this last parametrisation was chosen according to the IRN specifications of the dyads used in the experiments in the Main Text). Notice again the lack of responses out of the perceptual range of the model (i.e., for *T* > 15 ms).
(TIF)

S9 Fig Predicted latencies for other families of dyads. As in Fig 5k, strongly consonant dyads are represented with a green triangle, whilst strongly dissonant dyads are represented with a red triangle [6]. Dyad and experimental parameters were the same as in Fig 5k, with the following changes: a) lower-pitched dyads, with *f*_0_ = 100 Hz instead of 160 Hz; b) higher-pitched dyads: *f*_0_ = 200 Hz; c) *equal temperament* [6] was used instead of the *just intonation* to calculate the chromatic scale; d) dyads were generated using IRNs with 32 rather than 8 iterations. These additional results faithfully reproduce the effect of consonance on latency reported in Fig 5. Moreover, panels a) and b) show that the latency differences due to pitch change are smaller than the latency differences induced by dissonance. As in Fig 5, results were averaged across *N* = 60 runs of the model; error bars are standard errors.
(TIF)

S1 Text Attractor dynamics and pitch transitions. In this supplementary text we analyze the decoding process from a Dynamic System’s perspective and show how pitch transitions are represented in the phase map spanned by the dynamical variables of the model.
(PDF)

S2 Text Model predictions for other stimuli. In this supplementary text we consider the response of the model to stimuli that trigger both, an energy onset response and a pitch onset response.
(PDF)

S3 Text Supplementary methods. In this supplementary text we explain in more detail how the regularization of the model’s input, based on the summary autocorrelation function, is computed in our simulations.
(PDF)
