# Correction: Modeling and MEG evidence of early consonance processing in auditory cortex

**DOI:** 10.1371/journal.pcbi.1009694

**Published:** 2021-12-13

**Authors:** Alejandro Tabas, Martin Andermann, Valeria Schuberth, Helmut Riedel, Emili Balaguer-Ballester, André Rupp

There is an error in Fig 1. Please find the corrected Fig 1. below:

**Fig 1:**
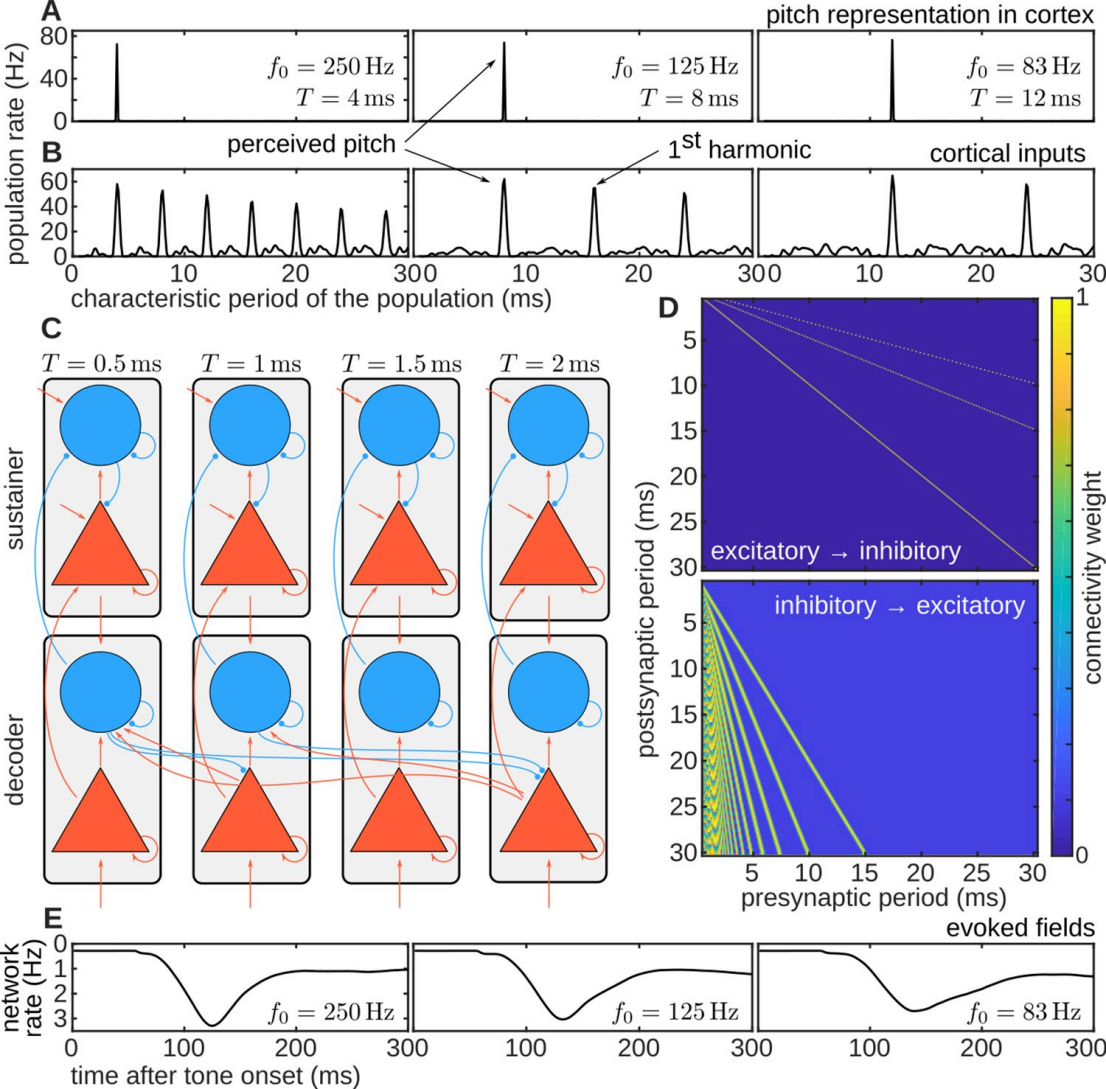
Basic schematics of the model. Architecture (C, D) and responses (A, B, E) of the model to three stimuli with different pitches. Stimulus used to produce the examples were iterated rippled noises with 16 iterations, bandpass filtered between 0.8 and 3.2 kHz, with three fundamental periods T = 4, 8, and 12 ms, corresponding to the three columns of the figure. (A) Excitatory population rate in the decoder (i.e., the time-average response for each of the excitatory ensembles in the decoder). The rate was averaged between 250 and 300 ms after the sound onset. The main peak of the population rate at the decoder represents stimulus pitch. (B) Excitatory population rate of the cortical input (i.e., the time-average response for each periodicity detector). As in panel A, the rate was averaged between 250 and 300 ms after sound onset. The first peak in this representation corresponds to the fundamental period of the stimulus; subsequent peaks correspond to its lower harmonics. (C) Model architecture. The model consists of two networks, each with 250 columns (grey rectangles). Each column comprises an excitatory (triangle) and an inhibitory (circle) ensemble, and represents a specific pitch value ranging from 1/(0.5 ms) = 2 kHz to 1/(30 ms) = 33.3 Hz. The bottom network is termed the decoder, and the top network is called the sustainer (see text). Red arrows between ensembles represent excitatory connections; blue lines ended in a circle denote inhibitory connections. (D) Connectivity weights between excitatory and inhibitory ensembles in the decoder network. (E) Decoder’s network rate (i.e., the average response across all the excitatory ensembles of the decoder network at each instant t), monotonically related to the auditory evoked fields. The y-axis was inverted for consistency with the standard representation of the evoked fields. The network rate peak latency correlates with the latency of the pitch onset response.
