# Supplementary figures and images for: Modeling and MEG evidence of early consonance processing in auditory cortex
Source: PLoS Comput Biol. 2019 Feb 28;15(2):e1006820. doi: 10.1371/journal.pcbi.1006820 (PMC6413961; doi:10.1371/journal.pcbi.1006820)

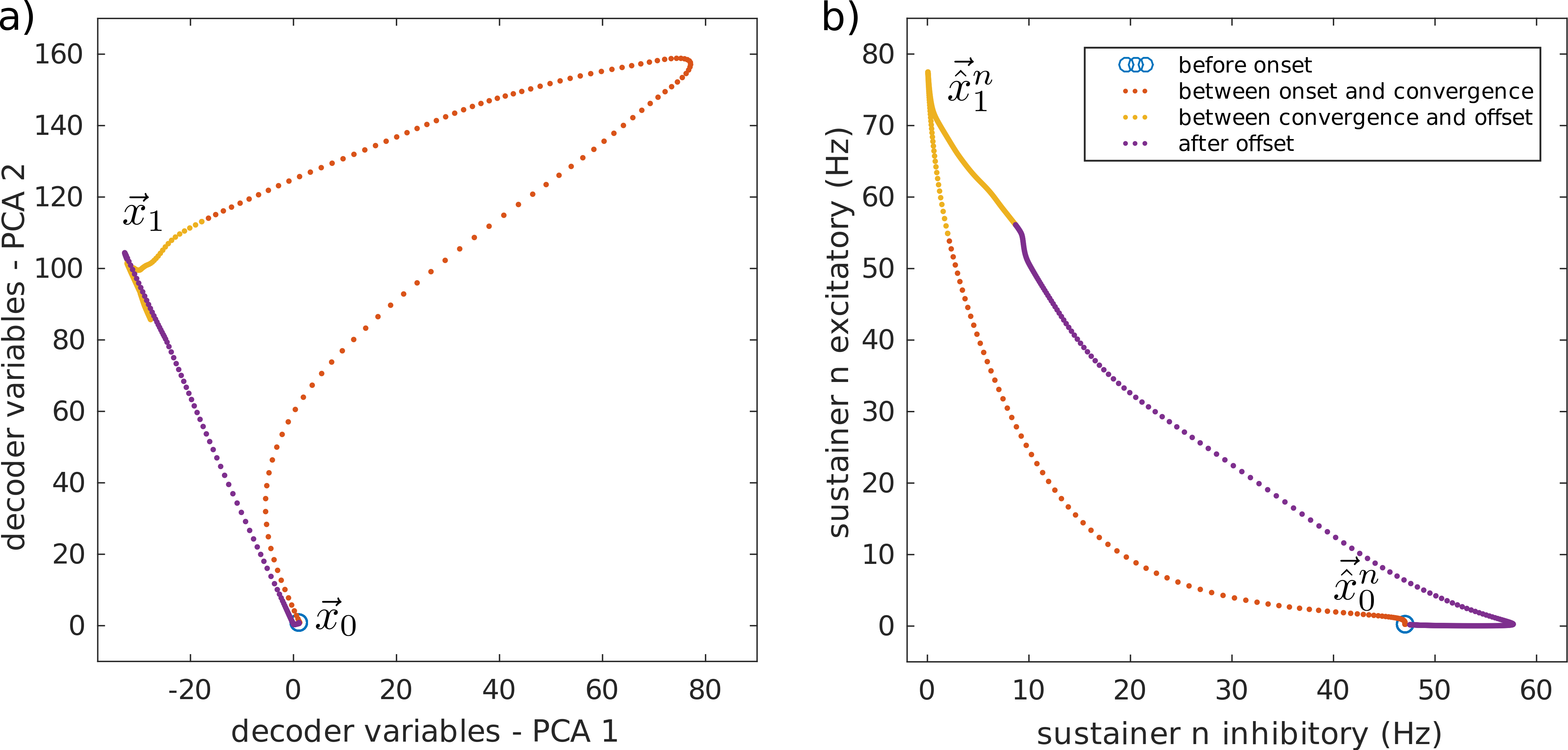

Supplement: S1 Fig — a) Two-dimensional projection of the state variables x→ during pitch processing using principal components analysis (PCA; see caption in S1 Video for details). The trajectory in the reduced space reveals key aspects of the onset and relaxation dynamics; the transition from x→0 to x→1 characterizes the POR. b) View of the two dimensions of the subsystem characterizing the decoded pitch n in the sustainer network (see section S1.2). Note that the relaxation dynamics of the sustainer network, corresponding to the transition from x→^1n to x→^0n, are much slower than the relaxation dynamics of the decoder network; resembling the sustained field offset delay [67]. See also the caption in S1 Video. (TIF) [file pcbi.1006820.s003.tif]

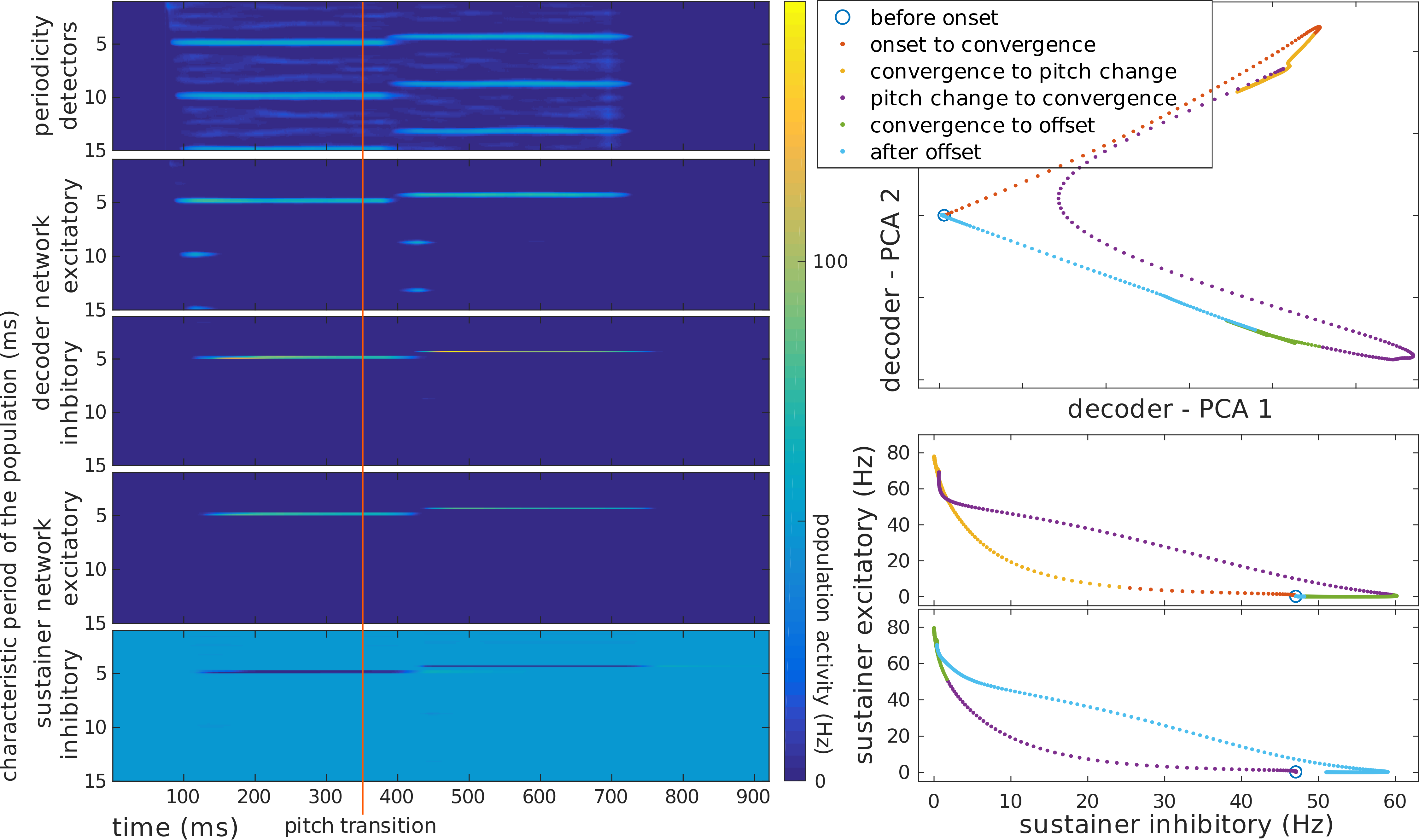

Supplement: S2 Fig — a) Response to pitch changes (see caption in Fig 2 in the Main Text for details). b)–c) Representation of the attractor dynamics of the model under pitch changes. Two colours were added to represent the new states in the system’s evolution: purple now represents the dynamics from the second stimulus onset to the new state of convergence, defined here as the state achieved 135 ms after the onset; green represents states between convergence and the second stimulus offset; and light blue represent the states during the relaxation dynamics after offset. The remaining colours are kept as in S1 Fig and S1 Video. Note that the transition from x→1 to x→2 elicits a new, second POR corresponding to the second stimulus. Stimuli were IRNs with the same specifications as in [9]; first tone had a fundamental frequency f0 = 200 Hz, second tone was two semitones higher than the first note, with f0 = 225 Hz. The pitch transition occurs 350 ms after the onset of the first tone (see arrow in the figure). (TIF) [file pcbi.1006820.s004.tif]

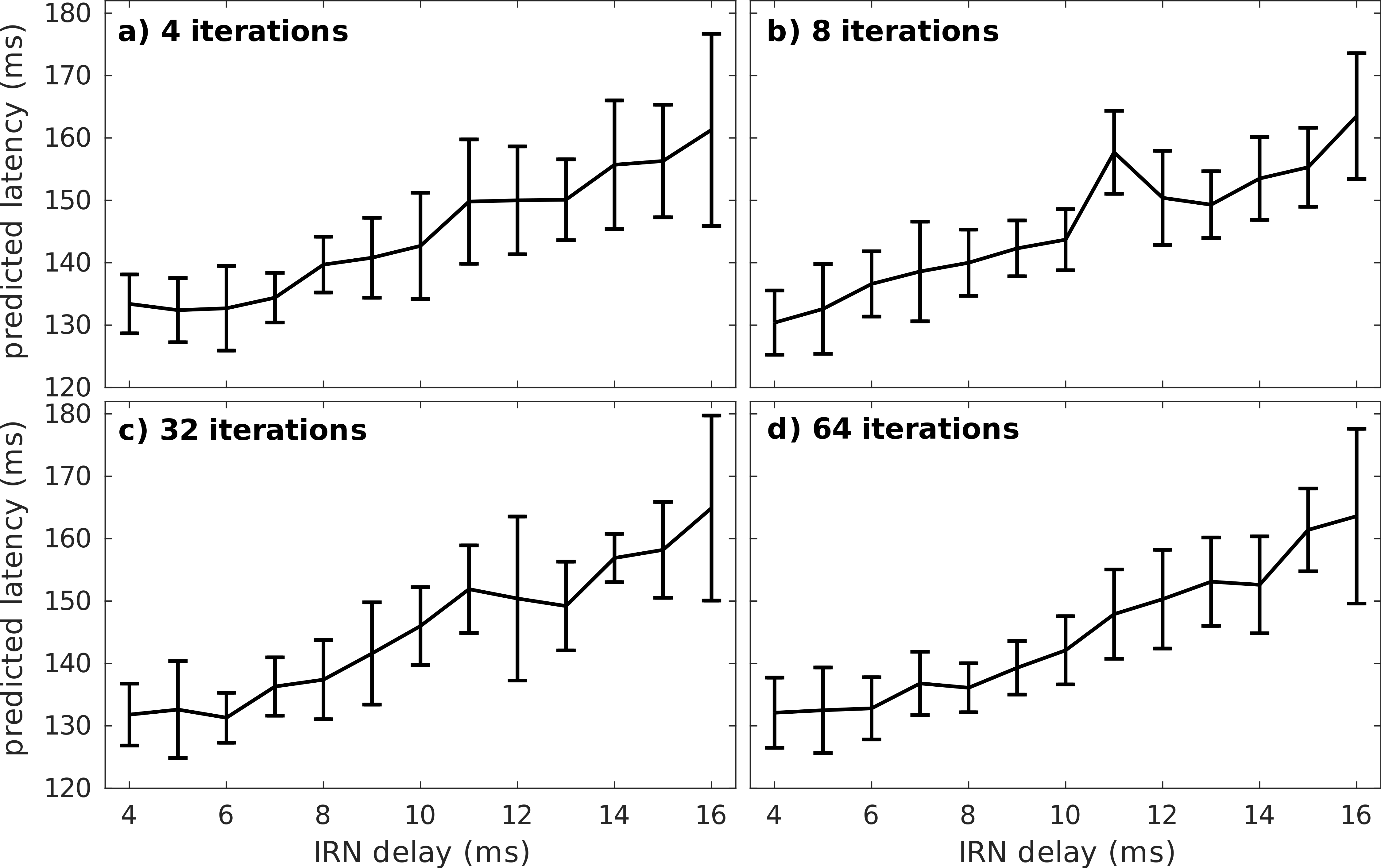

Supplement: S3 Fig — Except for the number of iterations of the IRNs and the number of runs used to obtain the results (in this case, N = 10) simulation parameters were the same as in Fig 3a; error bars are standard deviations. Although experimental data is not available for these stimuli, results faithfully replicate the trends reported in Fig 3a. (TIF) [file pcbi.1006820.s005.tif]

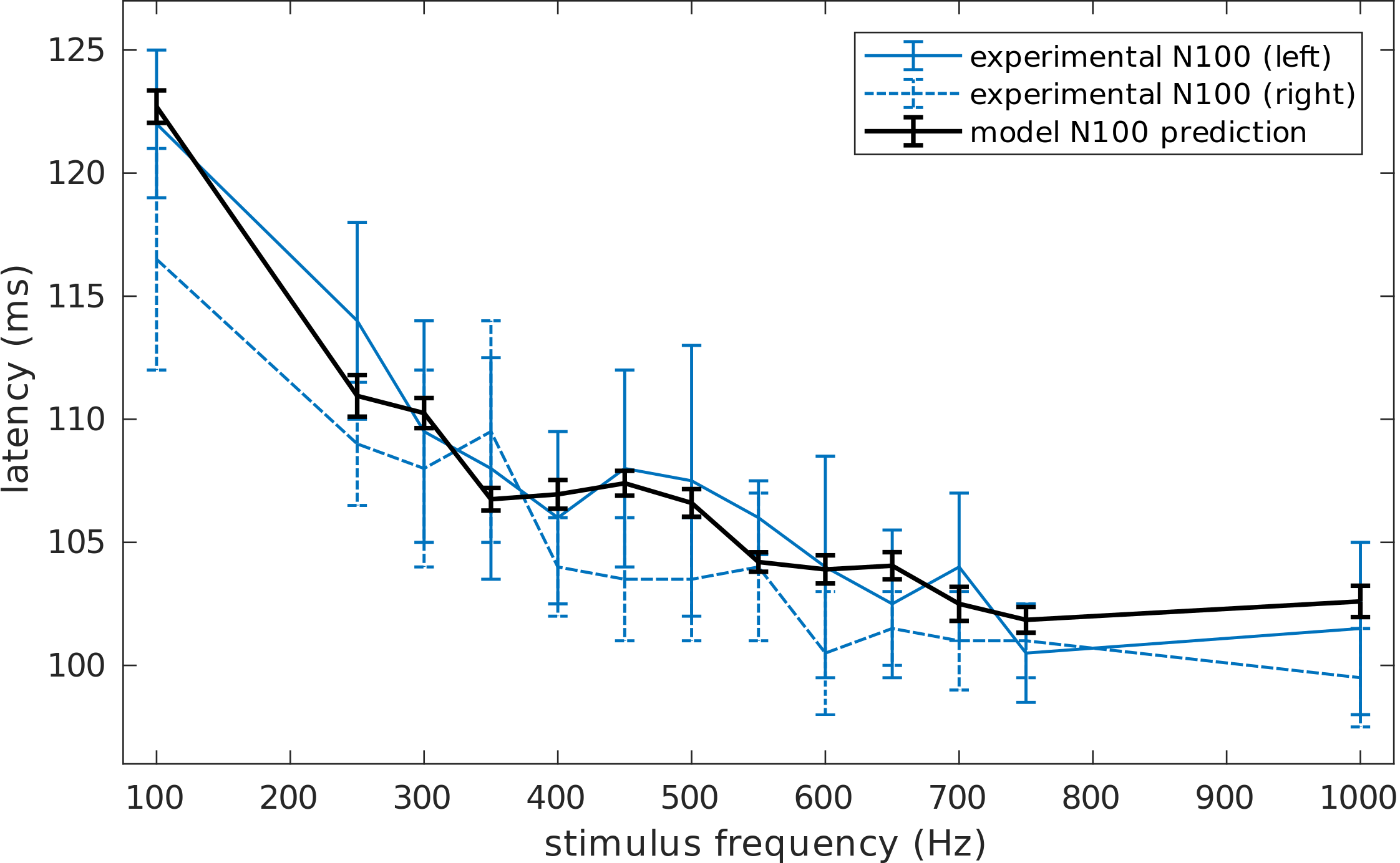

Supplement: S4 Fig — a) Simulated N100 latency values (black error bars) and N100 latency observations (blue error bars); the two experimental curves correspond to latency values observed in the right and left hemispheres. Predictions were averaged along N = 10 runs of the model; error bars are standard errors. Experimental data was taken from Roberts et al. [39], Fig 2. (TIF) [file pcbi.1006820.s006.tif]

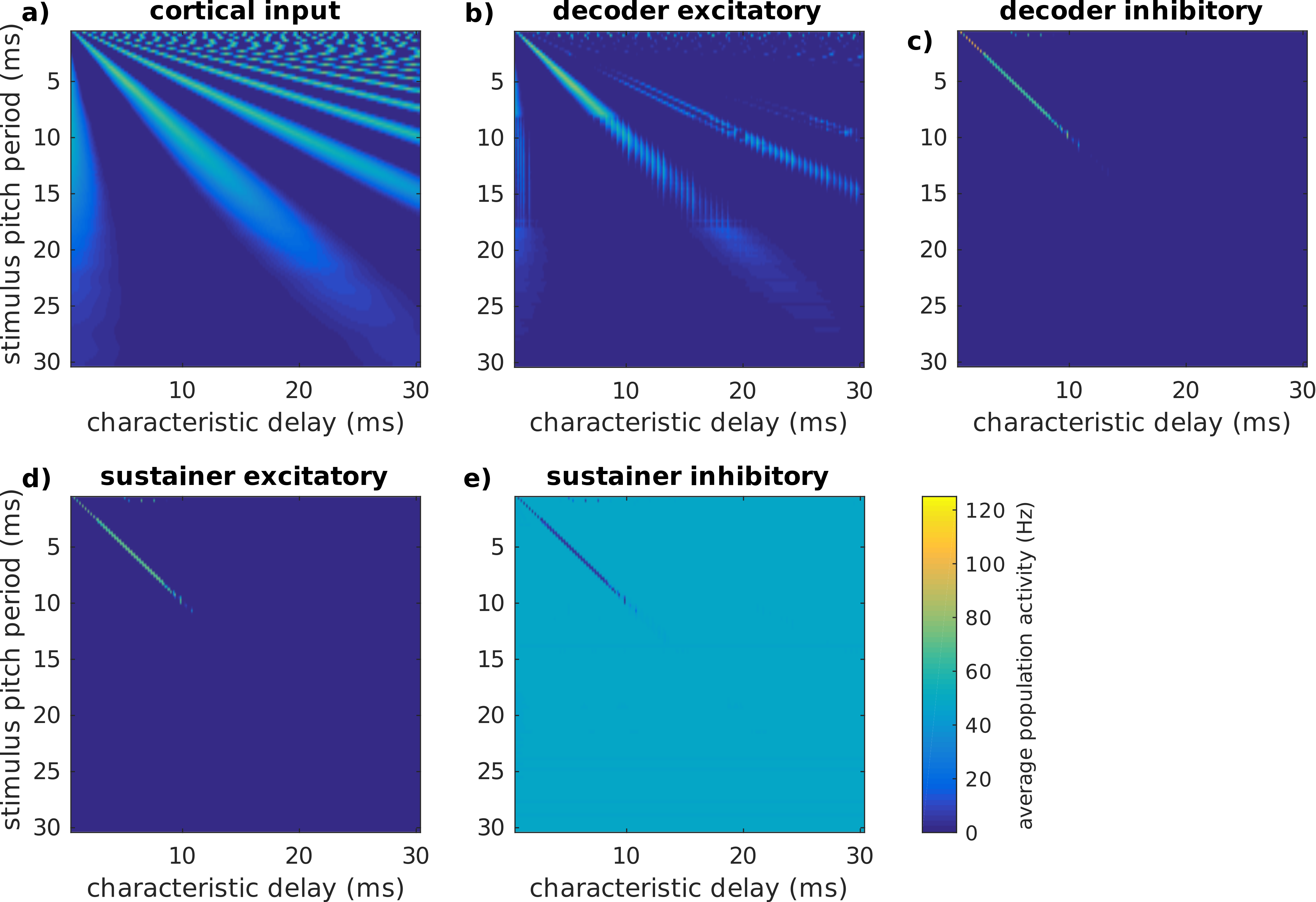

Supplement: S5 Fig — Averaged model responses to pure tones at different stages of the model: (a) periodicity detectors, (b/c) excitatory/inhibitory ensembles in the decoder, (d/e) excitatory/inhibitory ensembles in the sustainer. The decay of the responses under f ∼ 125Hz (or T ∼ 8 ms) is due to the lower-frequency limit of the peripheral model [25]. The Figure was produced using the same methodology as in Fig 3d–3h (see Main Text for details). (TIF) [file pcbi.1006820.s007.tif]

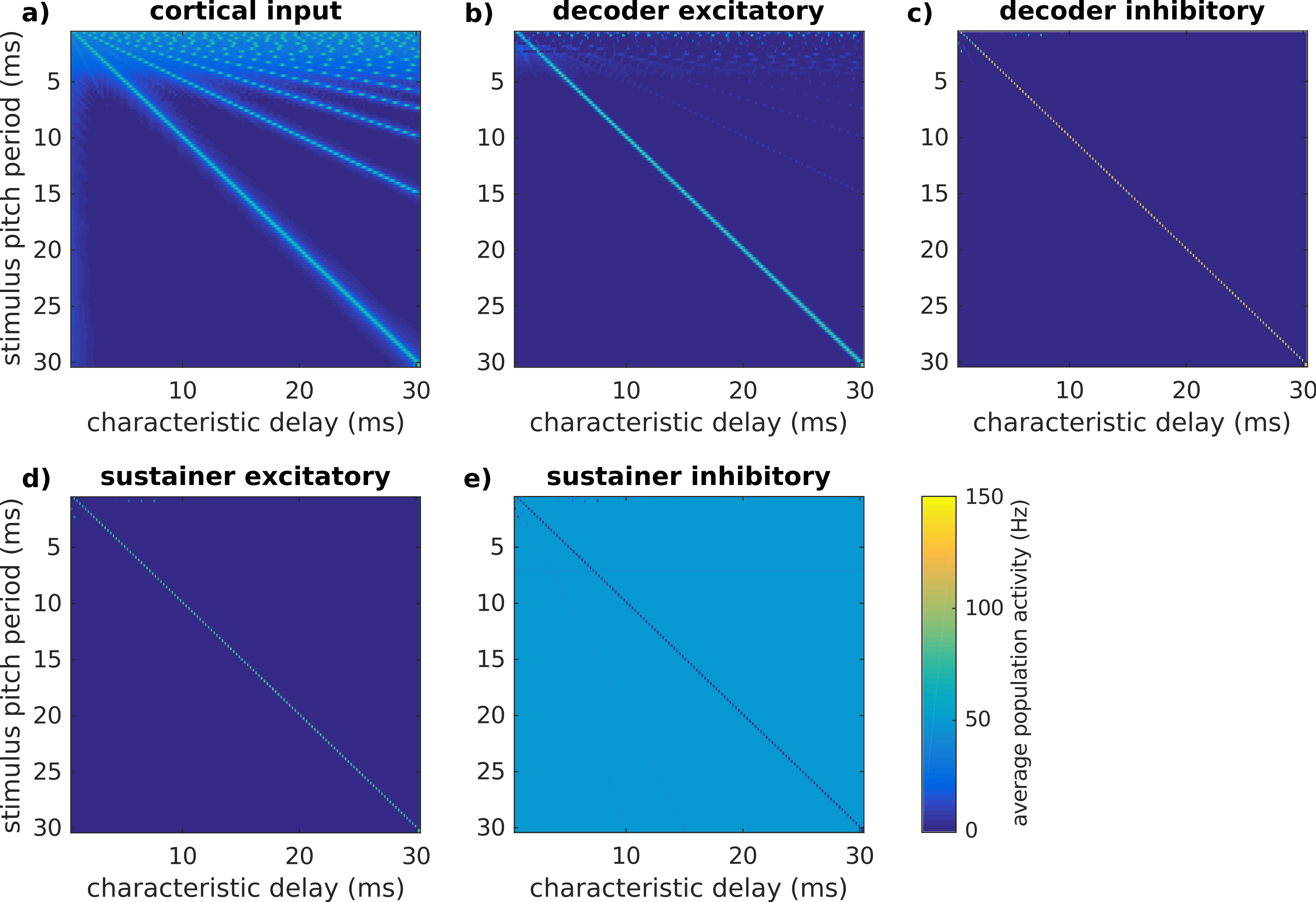

Supplement: S6 Fig — Click trains (generated as a train of Dirac deltas) elicit the same pitch sensation as a sine wave with period T equal to the interclick interval [42]. Colormaps show the averaged responses click trains at different stages of the model: (a) periodicity detectors, (b/c) excitatory/inhibitory ensembles in the decoder, (d/e) excitatory/inhibitory ensembles in the sustainer. Results are fully consistent with experimental observations [42]. (TIF) [file pcbi.1006820.s008.tif]

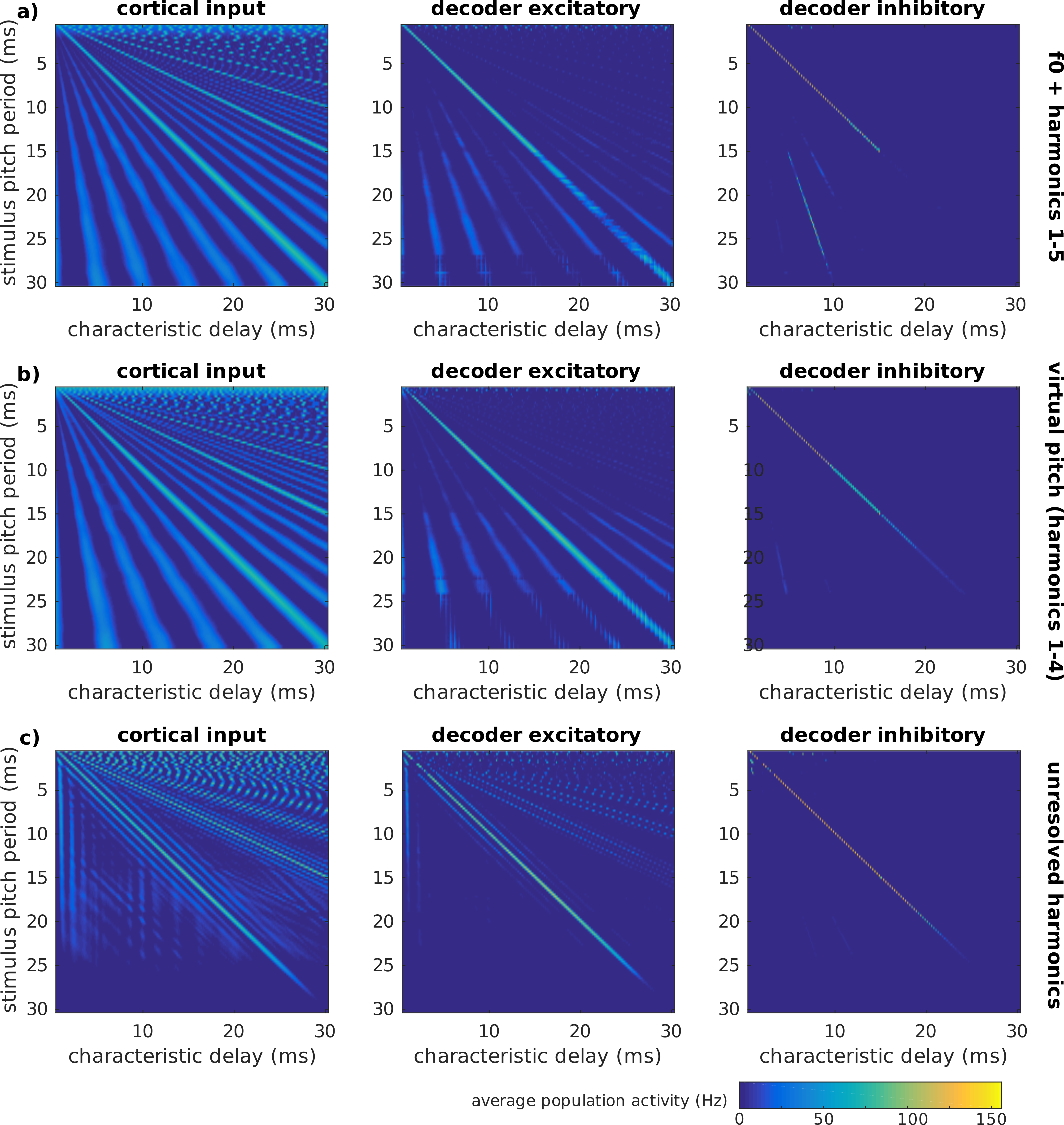

Supplement: S7 Fig — HCTs elicit the same pitch percept as a sine wave with the frequency of the fundamental of the complex, even if the fundamental itself is not comprised in the complex (known as the virtual pitch [41]). The figure shows the responses of the model for: a) HCTs formed by the fundamental and the first 5 higher harmonics; b) HCTs with a missing fundamental (comprising only by the first four higher harmonics); c) HCTs with a missing fundamental comprising harmonics that are not independently resolved in the cochlea (tones were generated as harmonic complexes with harmonics 1 to 50, bandpass filtered between 3.2 kHz and 5 kHz). Note that, since the model uses several peaks of the harmonic series to extract the pitch value from the representation in the periodicity detectors, the perceptual range of the model is limited to periods T < 15 ms. Averaged responses in the sustainer populations are precisely correlated with the responses in the inhibitory ensembles in the decoder (omitted here for simplicity). (TIF) [file pcbi.1006820.s009.tif]

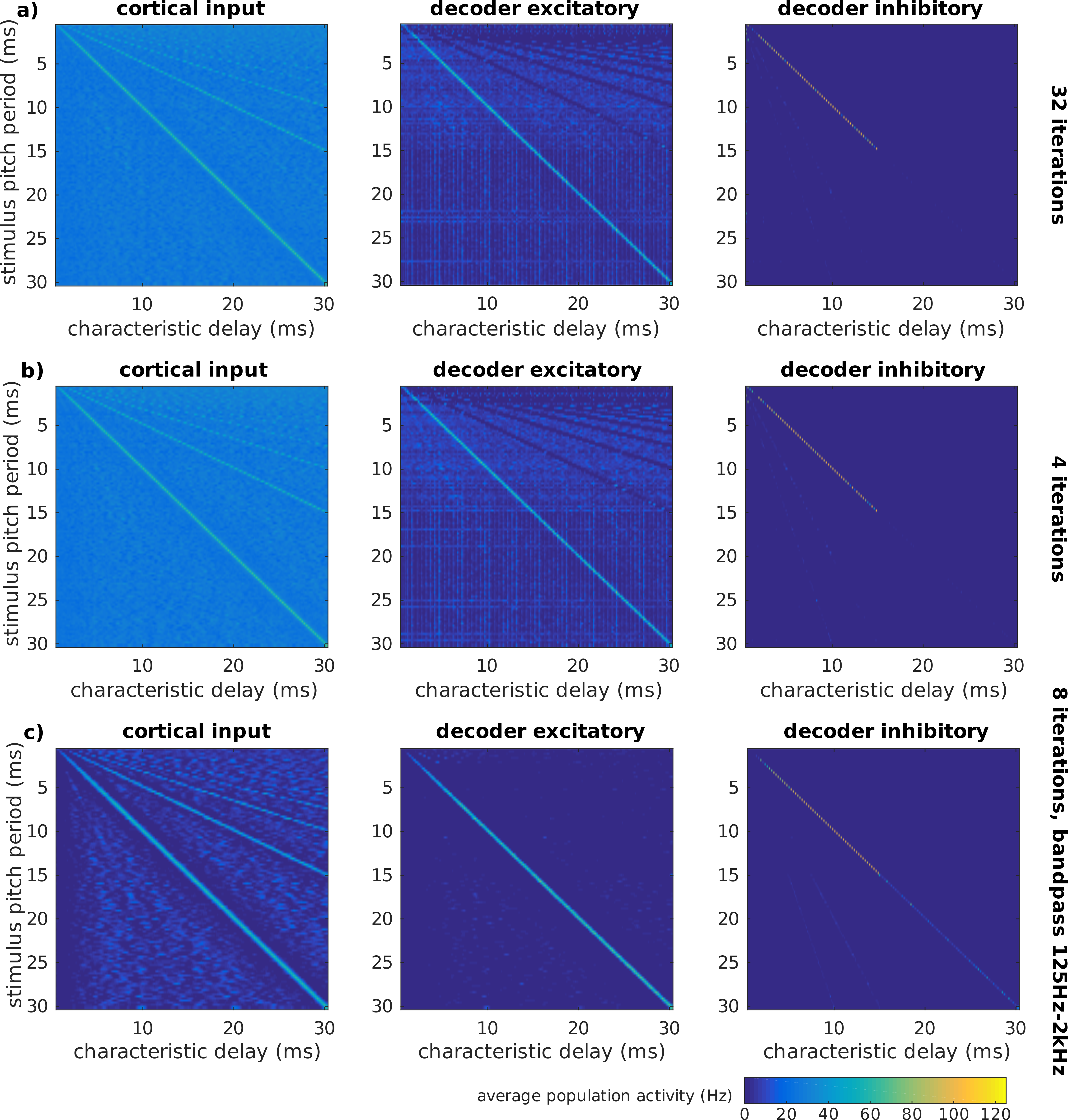

Supplement: S8 Fig — The figure shows the perceptual responses for additional classes of iterated rippled noises (IRN) with different parametrisations (see also Fig 3 in the Main Text): a) IRN with 32 iterations and no filtering; b) IRN with 4 iterations and no filtering; c) IRN with 8 iterations, bandpass filtered between 125 Hz and 2 kHz (this last parametrisation was chosen according to the IRN specifications of the dyads used in the experiments in the Main Text). Notice again the lack of responses out of the perceptual range of the model (i.e., for T > 15 ms). (TIF) [file pcbi.1006820.s010.tif]

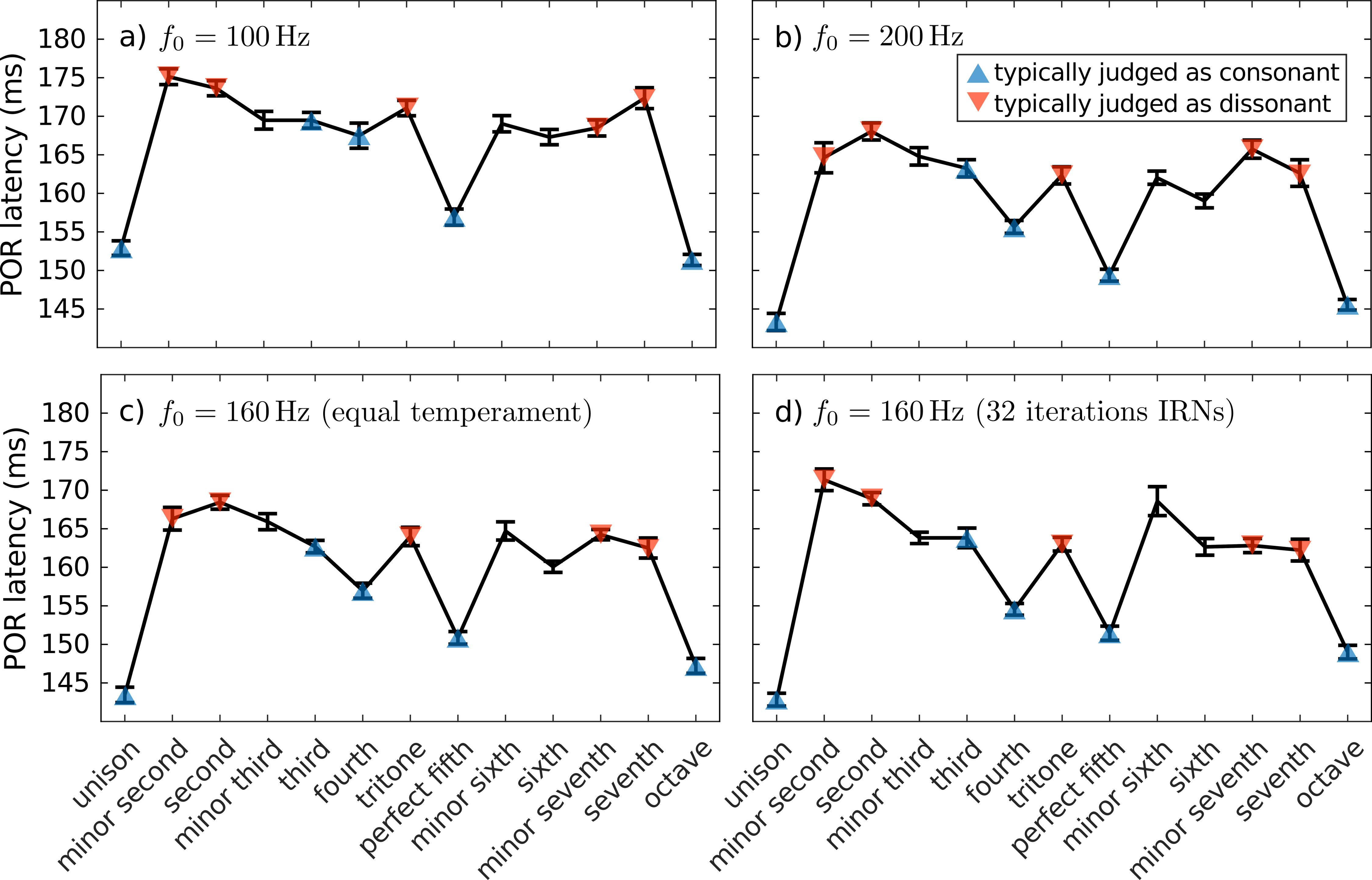

Supplement: S9 Fig — As in Fig 5k, strongly consonant dyads are represented with a green triangle, whilst strongly dissonant dyads are represented with a red triangle [6]. Dyad and experimental parameters were the same as in Fig 5k, with the following changes: a) lower-pitched dyads, with f0 = 100 Hz instead of 160 Hz; b) higher-pitched dyads: f0 = 200 Hz; c) equal temperament [6] was used instead of the just intonation to calculate the chromatic scale; d) dyads were generated using IRNs with 32 rather than 8 iterations. These additional results faithfully reproduce the effect of consonance on latency reported in Fig 5. Moreover, panels a) and b) show that the latency differences due to pitch change are smaller than the latency differences induced by dissonance. As in Fig 5, results were averaged across N = 60 runs of the model; error bars are standard errors. (TIF) [file pcbi.1006820.s011.tif]
