# Supplementary material for: Modeling and MEG evidence of early consonance processing in auditory cortex
Source: PLoS Comput Biol. 2019 Feb 28;15(2):e1006820. doi: 10.1371/journal.pcbi.1006820 (PMC6413961; doi:10.1371/journal.pcbi.1006820)
Supplement: S1 Text — In this supplementary text we analyze the decoding process from a Dynamic System’s perspective and show how pitch transitions are represented in the phase map spanned by the dynamical variables of the model. (PDF) [file pcbi.1006820.s012.pdf]

Supplementary materials to "Modeling and MEG evidence of early consonance processing in the cortical pitch response"

Alejandro Tabas*, Martin Andermann*, Valeria Schuberth, Helmut Riedel
Emili Balaguer-Ballester+, & André Rupp+

Text S1: Attractor dynamics and pitch transitions

The behavior of the decoder network can be characterized by a dynamical system with variables $\vec{x} = \{H_n^e, H_n^i, S_n^{\mathrm{AMPA}}, S_n^{\mathrm{NMDA}}, S_n^{\mathrm{GABA}}\}^{n=1\ldots N}$ (see Methods). In absence of input drive, the system presents a single stable state around the origin $\vec{x} = \vec{x}_0 \simeq 0$.

Cortical inputs change the stability properties of the system. An excitatory cortical input shifts this equilibrium state towards a new attractor state termed here $\vec{x}_{\mathrm{input}}$, where the excitatory populations represent the input activity. If the input presents a harmonic structure, the system converges to a second equilibrium state we termed $\vec{x}_1$, characterized by excitatory and inhibitory activation at the column encoding the stimulus pitch (Figure S1a, Video S1).

We identify the POR as the neuromagnetic representation of this two-stage transition: the build up of the transient corresponds to the transition $\vec{x}_0 \to \vec{x}_{\mathrm{input}}$; the POR peaks shortly after the onset of the decoding process, characterized by the transition $\vec{x}_{\mathrm{input}} \to \vec{x}_1$ (see Figure S1a). This identification onnects the POR latency with the time necessary to trigger the $\vec{x}_{\mathrm{input}} \to \vec{x}_1$ transition; thus, the POR latency is informative of the convergence time in the decoder network (see also Video S1).

The role of the sustainer network is to modulate the dynamic properties of the decoder in order to prevent the spurious reversed transition $\vec{x}_1 \to \vec{x}_{\mathrm{input}}$ and subsequent oscillations that are not observed in the recorded magnetic field responses.

The sustainer network dynamics (shown in Figure S1b and in Video S1) is much simpler than the decoder dynamics; it consists of $N = 250$ uncoupled dynamical systems, one per column, with 5 variables each $\hat{x}^n = \left\{\hat{H}_n^e, \hat{H}_n^i, \hat{S}_n^{\mathrm{AMPA}}, \hat{S}_n^{\mathrm{NMDA}}, \hat{S}_n^{\mathrm{GABA}}\right\}$. At rest, the decoder's independent variables lie in equilibrium states $\vec{\hat{x}}^n = \vec{\hat{x}}_0$ characterized by a strong activation in the inhibitory population and a null activation in the excitatory ensemble of each column n.

Combined excitatory and inhibitory input from the decoder network to a given column $\vec{\hat{x}}^n$ of the sustainer network causes inhibition to drop and excitation to rise, switching the ensembles of the column to a new state $\vec{\hat{x}}_1$ termed here *sustained state*. Subsequently, top-down efferents from the sustainer network lock to the pitch-selective dynamics of the decoder, strengthening the attractor properties of the decoder network state $\vec{x}_1$ and turning it to a robust stable equilibrium state (see Figure S1b).

When the cortical input is switched off (the behavior of the model under pitch changes is addressed in Figure S2), excitatory activity in the decoder drops, removing the excitatory input at the sustainer column $\vec{\hat{x}}^n$, which returns to its resting state $\vec{\hat{x}}_0$. As a result, the sustainer column stops modulating the dynamics of the decoder and the state $\vec{x}_1$ becomes, once again, unstable. Thus, the decoder state slowly relaxes back to the origin state $\vec{x}_0$ (Figure S1b, Video S1).

References

[1] R. Näätänen and T. Picton, "The N1 wave of the human electric and magnetic response to sound: a review and an analysis of the component structure," *Psychophysiology*, vol. 24, no. 4, pp. 375–425, 1987.

[2] K. Krumbholz, R. D. Patterson, A. Seither-Preisler, C. Lammertmann, and B. Lütkenhöner, "Neuromagnetic evidence for a pitch processing center in Heschl's gyrus," *Cerebral Cortex*, vol. 13, no. 7, pp. 765–772, 2003.

[3] A. Seither-Preisler, R. Patterson, K. Krumbholz, S. Seither, and B. Lütkenhöner, "Evidence of pitch processing in the N100m component of the auditory evoked field," *Hearing Research*, vol. 213, no. 1-2, pp. 88–98, 2006.

[4] M. Andermann, R. D. Patterson, C. Vogt, L. Winterstetter, and A. Rupp, "Neuromagnetic correlates of voice pitch, vowel type, and speaker size in auditory cortex," *NeuroImage*, vol. 158, pp. 79–89, 2017.

[5] R. Meddis and L. O'Mard, "A unitary model of pitch perception," *The Journal of the Acoustical Society of America*, vol. 102, no. 3, pp. 1811–1820, 1997.

[6] R. Meddis and L. P. O'Mard, "Virtual pitch in a computational physiological model," *The Journal of the Acoustical Society of America*, vol. 120, no. 6, p. 3861, 2006.

[7] E. Balaguer-Ballester, S. L. Denham, and R. Meddis, "A cascade autocorrelation model of pitch perception," *The Journal of the Acoustical Society of America*, vol. 124, no. 4, pp. 2186–95, 2008.

[8] J. F. Brugge, K. V. Nourski, H. Oya, R. a. Reale, H. Kawasaki, M. Steinschneider, and M. a. Howard, "Coding of repetitive transients by auditory cortex on Heschl's gyrus," *Journal of neurophysiology*, vol. 102, no. 4, pp. 2358–74, 2009.

[9] A. de Cheveigné, "Pitch Perception Models," in *Pitch: Neural Coding and Perception* (C. J. Plack, R. R. Fay, A. J. Oxenham, and A. N. Popper, eds.), ch. 6, pp. 169–233, Springer New York, 2005.

[10] T. D. Griffiths, S. Uppenkamp, I. Johnsrude, O. Josephs, and R. D. Patterson, "Encoding of the temporal regularity of sound in the human brainstem," *Nature Neuroscience*, vol. 4, no. 6, pp. 633–7, 2001.

[11] H. Penagos, J. R. Melcher, and A. J. Oxenham, "A neural representation of pitch salience in nonprimary human auditory cortex revealed with functional magnetic resonance imaging," *The Journal of Neuroscience*, vol. 24, no. 30, pp. 6810–5, 2004.

[12] K.-F. Wong and X.-J. Wang, "A recurrent network mechanism of time integration in perceptual decisions," *The Journal of Neuroscience*, vol. 26, no. 4, pp. 1314–1328, 2006.
