# Supplementary material for: Modeling and MEG evidence of early consonance processing in auditory cortex
Source: PLoS Comput Biol. 2019 Feb 28;15(2):e1006820. doi: 10.1371/journal.pcbi.1006820 (PMC6413961; doi:10.1371/journal.pcbi.1006820)
Supplement: S2 Text — In this supplementary text we consider the response of the model to stimuli that trigger both, an energy onset response and a pitch onset response. (PDF) [file pcbi.1006820.s013.pdf]

Supplementary materials to "Modeling and MEG evidence of early consonance processing in the cortical pitch response"

Alejandro Tabas*, Martin Andermann*, Valeria Schuberth, Helmut Riedel
Emili Balaguer-Ballester+, & André Rupp+

Text S2: Model predictions for other stimuli

Although our model successfully explained the morphology and latency of the POR elicited by stimuli based on iterated rippled noise, the extrapolation of these results to additional stimulus types is limited by experimental constraints. Stimuli not based on IRN do not have an energy-matched counterpart; thus, their elicited POR cannot be disentangled from the rest of subcomponents on the N100 complex [1].

Here, we tackle this problem by expressing the N100 latency as an average between the latency of the POR and an energy onset response (EOR). This simple model allows us to correct our POR predictions by assuming that the latency of the EOR is always 100 ms [2], that both transients have the same amplitude, and that their generators are equally distant from the N100 equivalent dipole. Corrected values are shown in Figures S3.

Despite the success of the simple model of the N100 introduced above, harmonic complex tones elicit N100 transients with shorter peak latencies than the latency we assumed for the EOR [3]. Future work should approach this issue by providing for a detailed model of the EOR and other (timbre-related) transients contributing to the N100 [4].

References

[1] R. Näätänen and T. Picton, "The N1 wave of the human electric and magnetic response to sound: a review and an analysis of the component structure," *Psychophysiology*, vol. 24, no. 4, pp. 375–425, 1987.

[2] K. Krumbholz, R. D. Patterson, A. Seither-Preisler, C. Lammertmann, and B. Lütkenhöner, "Neuromagnetic evidence for a pitch processing center in Heschl's gyrus," *Cerebral Cortex*, vol. 13, no. 7, pp. 765–772, 2003.

[3] A. Seither-Preisler, R. Patterson, K. Krumbholz, S. Seither, and B. Lütkenhöner, "Evidence of pitch processing in the N100m component of the auditory evoked field," *Hearing Research*, vol. 213, no. 1-2, pp. 88–98, 2006.

[4] M. Andermann, R. D. Patterson, C. Vogt, L. Winterstetter, and A. Rupp, "Neuromagnetic correlates of voice pitch, vowel type, and speaker size in auditory cortex," *NeuroImage*, vol. 158, pp. 79–89, 2017.
