# Supplementary material for: Modeling and MEG evidence of early consonance processing in auditory cortex
Source: PLoS Comput Biol. 2019 Feb 28;15(2):e1006820. doi: 10.1371/journal.pcbi.1006820 (PMC6413961; doi:10.1371/journal.pcbi.1006820)
Supplement: S3 Text — In this supplementary text we explain in more detail how the regularization of the model’s input, based on the summary autocorrelation function, is computed in our simulations. (PDF) [file pcbi.1006820.s014.pdf]

Supplementary materials to "Modeling and MEG evidence of early consonance processing in the cortical pitch response"

Alejandro Tabas*, Martin Andermann*, Valeria Schuberth, Helmut Riedel
Emili Balaguer-Ballester+, & André Rupp+

Text S3: Supplementary Methods - regularisation of the input to the decoder network

The output of the array of coincidence detectors, based on the summary autocorrelation function (SACF) [1–3] for each characteristic period $A_n(t)$, was regularised $A_n(t) \to \hat{A}_n(t)$ through a four-step procedure in order to reduce its dependence with stimulus intensity levels and to minimize strong signal-to-noise variations with different stimulus timbres.

In the first step, $A_n(t)$ is low-pass filtered using a $\tau = 20\,\mathrm{ms}$ leaky-integrator that reflects the degradation of phase-locked activity over 50 Hz in auditory cortex [4]. The low-pass $A_n(t)$ is then normalised by dividing the overall signal by the low-passed summary self-correlation of the auditory nerve activity [5]. A fixed baseline $b_0 = 0.35$ is then subtracted from the overall function. Baseline was chosen such that a white noise stimulus elicits no activation, in agreement with fMRI studies that reported pitch-selective activation in inferior colliculus [6, 7].

Last, the normalized baseline-corrected $A_n(t)$ is rescaled to firing rate units (Hz) by a constant factor $A_0 = 75\,\mathrm{Hz}/(1 - b_0)$ that yields typical input activation peak values of $\sim 60\,\mathrm{Hz}$, in agreement with previous cortical models of perceptual integration [8]. Average activation in the regularised SACF associated to dyads was around half of the size of the average activation elicited by single IRNs (with the only exception of the unison, which was essentially a single IRN). Thus, in order to preserve the same $\sim 60\,\mathrm{Hz}$ peak activation also in dyads we used a doubled rescaling factor ($A_0^{\mathrm{dyads}} = 2\,A_0$).

References

[1] R. Meddis and L. O'Mard, "A unitary model of pitch perception," *The Journal of the Acoustical Society of America*, vol. 102, no. 3, pp. 1811–1820, 1997.

[2] R. Meddis and L. P. O'Mard, "Virtual pitch in a computational physiological model," *The Journal of the Acoustical Society of America*, vol. 120, no. 6, p. 3861, 2006.

[3] E. Balaguer-Ballester, S. L. Denham, and R. Meddis, "A cascade autocorrelation model of pitch perception," *The Journal of the Acoustical Society of America*, vol. 124, no. 4, pp. 2186–95, 2008.

[4] J. F. Brugge, K. V. Nourski, H. Oya, R. a. Reale, H. Kawasaki, M. Steinschneider, and M. a. Howard, "Coding of repetitive transients by auditory cortex on Heschl's gyrus," *Journal of neurophysiology*, vol. 102, no. 4, pp. 2358–74, 2009.

[5] A. de Cheveigné, "Pitch Perception Models," in *Pitch: Neural Coding and Perception* (C. J. Plack, R. R. Fay, A. J. Oxenham, and A. N. Popper, eds.), ch. 6, pp. 169–233, Springer New York, 2005.

[6] T. D. Griffiths, S. Uppenkamp, I. Johnsrude, O. Josephs, and R. D. Patterson, "Encoding of the temporal regularity of sound in the human brainstem," *Nature Neuroscience*, vol. 4, no. 6, pp. 633–7, 2001.

[7] H. Penagos, J. R. Melcher, and A. J. Oxenham, "A neural representation of pitch salience in nonprimary human auditory cortex revealed with functional magnetic resonance imaging," *The Journal of Neuroscience*, vol. 24, no. 30, pp. 6810–5, 2004.

[8] K.-F. Wong and X.-J. Wang, "A recurrent network mechanism of time integration in perceptual decisions," *The Journal of Neuroscience*, vol. 26, no. 4, pp. 1314–1328, 2006.
